# Goal-setting mechanisms in educational management: a psychological perspective on student and teacher behavior

**DOI:** 10.3389/fpsyg.2025.1680752

**Published:** 2026-01-30

**Authors:** Yizhuo Wang, Shuzhu Tang, Yang Meng

**Affiliations:** Changchun Humanities and Sciences College, School of Social Welfare, Changchun, China

**Keywords:** educational management, goal-setting mechanisms, psychological mediating mechanisms, student behavior, teacher behavior

## Abstract

**Objective:**

To explore the psychological mechanisms and behavioral impacts of goal-setting mechanisms in educational management.

**Methods:**

A cross-sectional survey was conducted with 1,247 students and 358 teachers from 15 schools; data were analyzed using structural equation modeling, multilevel regression, and mediation effect testing.

**Results:**

Goal-setting mechanisms positively affected student learning behavior (*β* = 0.42, *p* < 0.001) and teacher teaching behavior (*β* = 0.38, *p* < 0.001); learning motivation and self-efficacy played a chain mediating role in students (indirect effect=0.21, 95%CI[0.15,0.28]), while professional identity and teaching efficacy mediated in teachers (indirect effect=0.19, 95%CI[0.12,0.26]); organizational support significantly moderated these relationships.

**Conclusion:**

Scientific goal-setting promotes positive teaching and learning behaviors through psychological pathways, providing insights for educational management.

## 1 Introduction

Goal-setting, as a core element of educational management, directly relates to educational quality and individual development. Locke and Latham’s goal-setting theory indicates that clear, challenging goals can significantly improve individual performance, a theory that has been widely validated in the educational field (Wynarczuk et al., 2025). Recent meta-analyses examining goal-setting interventions across educational contexts have demonstrated moderate to large effects on academic achievement and self-regulated learning behaviors (Ashraf, 2022). Research has also demonstrated that even brief psychological interventions targeting goal-related processes can meaningfully influence educational performance outcomes (Serra-Garcia et al., 2020). However, existing research predominantly focuses on the direct effects of goal-setting on performance outcomes, with relatively insufficient exploration of the underlying psychological mechanisms through which these effects occur. In recent years, research grounded in self-determination theory has revealed that the effectiveness of goal-setting largely depends on the degree to which individual psychological needs for autonomy, competence, and relatedness are satisfied (Guo et al., 2025). Meanwhile, the special nature of educational contexts causes students and teachers to exhibit different psychological response patterns in goal-setting processes, yet few studies have examined these populations simultaneously within an integrated theoretical framework.

Although some studies have explored the impact of goal-setting on learning motivation or teaching efficacy independently, there lacks a systematic theoretical framework to explain how goal-setting mechanisms influence behavioral performance through psychological pathways. Systematic reviews of educational goal-setting reveal that while motivational benefits are well-documented, the specific mediating roles of intrinsic motivation, self-efficacy, and professional identity remain insufficiently specified, particularly in non-Western educational systems. Based on this research gap, this study proposes the hypothesis that goal-setting mechanisms promote student learning behavior and teacher teaching behavior by influencing individual intrinsic motivation, self-efficacy, and other psychological factors.

In this study, “educational management” refers to the systematic organizational processes through which schools establish, communicate, and support goal achievement for both learning outcomes (students) and instructional practices (teachers). While educational managers—including school administrators and department heads—design and implement goal-setting mechanisms, our research focuses on examining the psychological and behavioral responses of the two primary stakeholder groups affected by these management practices: students as learners and teachers as instructional professionals. This dual focus allows us to understand how organizational goal-setting systems differentially influence behavior across distinct educational roles, providing comprehensive guidance for administrators who must simultaneously manage goals for multiple stakeholder groups.

This study contributes to the literature by: (1) simultaneously testing chain mediation models across student and teacher populations, addressing calls for more integrative research examining goal-setting across the educational ecosystem; (2) employing a large-scale multi-regional sample (*N* = 1,605) from 15 schools to enhance generalizability; (3) examining organizational support as a moderating variable, extending goal-setting theory’s contextual boundary conditions in educational management practice; and (4) specifying the psychological pathways through which organizational goal management translates into behavioral outcomes. While goal-setting research in education has traditionally focused on student achievement outcomes, teachers represent a critical yet understudied population whose goal-directed behaviors directly shape educational quality. Including both populations allows us to test whether goal-setting mechanisms operate through similar or distinct psychological pathways across educational roles, and to examine how organizational goal management systems must be differentiated to address the unique motivational structures of learners versus professionals.

## 2 Theoretical framework and hypotheses

### 2.1 Core elements of goal-setting theory in educational management

Goal-setting theory, as an important theoretical foundation in organizational behavior and educational psychology, demonstrates unique mechanisms of action in educational management practice. The goal-setting theory proposed by Locke and Latham emphasizes that goal clarity, challenge, acceptance, and feedback mechanisms are key factors affecting individual performance, a theoretical framework that has been widely validated and applied in the educational field. Goal clarity requires educational managers to set specific, measurable learning and teaching objectives, enabling students and teachers to clearly understand the expected standards to be achieved. Challenging goals need to set appropriate difficulty levels within the individual’s capability range, both avoiding motivational deficiency caused by overly simple goals and preventing frustration caused by excessively high goals.

Goal acceptance reflects the degree of individual recognition of goals, which directly affects the depth of goal internalization and the intensity of behavioral commitment. In educational contexts, students’ acceptance of learning goals is often influenced by multiple factors including teacher guidance methods, peer influence, and personal values. Teachers’ acceptance of teaching goals is closely related to professional development needs, organizational cultural atmosphere, and institutional support. The feedback mechanism, as an important component of goal-setting, helps individuals adjust behavioral strategies and effort directions through continuous information feedback. Effective feedback includes not only outcome feedback but more importantly process feedback, enabling learners and teachers to identify problems and make improvements in a timely manner.

### 2.2 Psychological theoretical foundation of student and teacher behavior

The psychological theoretical foundation of student and teacher behavior is built upon diversified motivation theories and cognitive theories, with self-determination theory and social cognitive theory providing important insights for understanding behavioral patterns in educational contexts. These four dimensions—clarity, challenge, acceptance, and feedback—were selected based on their theoretical grounding and empirical validation in educational settings. Goal clarity provides cognitive foundation for what needs accomplishing; challenge level determines motivational activation; acceptance ensures psychological ownership; and feedback enables continuous adjustment, collectively capturing effective goal-setting’s essential components that distinguish educational contexts from purely performance-oriented organizational settings (Liu and Yu, 2025). Student learning behavior is often driven by autonomy needs; when learning goals align with personal interests and values, students are more likely to demonstrate sustained learning engagement and deep cognitive processing. Competence needs are reflected in students’ confidence and sense of achievement in mastering learning content; appropriately challenging tasks can enhance students’ self-efficacy, thereby promoting positive learning behavioral performance.

The psychological foundation of teacher behavior is more reflected in the interaction between professional identity and teaching efficacy. Professional identity reflects teachers’ value cognition and emotional attachment to the education profession, and the strength of this identity directly affects teachers’ level of investment and innovative motivation in teaching activities (Mahere, 2025). Research on sustainable higher education management has highlighted that career drivers of academic staff, including professional identity and value alignment, significantly influence teachers’ goal-directed behaviors and long-term commitment to educational excellence (Dehtjare and Uzule, 2023). Teaching efficacy, as teachers’ beliefs about their teaching abilities, not only affects the selection and implementation of teaching strategies but more importantly affects persistence and adaptability when facing teaching challenges. High-efficacy teachers are more willing to try new teaching methods, show higher expectations for students, and demonstrate stronger resilience when encountering difficulties (Li and Guo, 2024).

The observational learning and self-regulation mechanisms in social cognitive theory provide another important perspective for understanding the formation and change of teacher-student behavior. Students form their own behavioral norms and learning habits by observing teachers’ behavioral patterns and peers’ learning strategies. Teachers similarly continuously adjust and optimize their teaching behaviors by observing excellent colleagues’ teaching practices and student feedback (Jing, 2024). Within our theoretical model, self-determination theory (SDT) and social cognitive theory (SCT) serve complementary functions in explaining the goal-setting to behavior pathway. SDT explains motivation initiation through basic psychological needs satisfaction, accounting for how goals become internalized, while SCT addresses behavior maintenance through efficacy beliefs and self-regulation processes. SDT frameworks inform the learning motivation (LM) and professional identity (PI) pathways—both reflecting satisfaction of intrinsic psychological needs—while SCT principles underlie self-efficacy (SE) and teaching efficacy (TE) constructs, representing confidence in behavioral execution capabilities. This theoretical integration captures both the motivational initiation phase and the behavioral execution phase of goal-directed action (Jaarsveld et al., 2025).

The synergy between goal-setting theory and SDT is theoretically grounded: goal-setting provides the structural framework—including clarity, challenge level, and feedback mechanisms—that creates environmental conditions enabling SDT’s basic psychological needs (autonomy, competence, relatedness) to be satisfied. When goals are self-concordant and presented with autonomy support, they activate intrinsic motivation by satisfying autonomy needs; when goals provide appropriate challenge and specific success criteria, they enhance competence perceptions and self-efficacy by providing clear pathways to capability demonstration. This integration addresses a gap in the literature where goal-setting theory and SDT have typically been examined separately rather than as complementary frameworks explaining different phases of the motivation-to-behavior process. Our model proposes that effective goal-setting in educational management creates the conditions necessary for psychological need satisfaction, which in turn activates the motivational and efficacy pathways that drive behavioral engagement and performance.

### 2.3 Psychological mediating mechanisms of goal-setting’s impact on behavior

**Figure 1:**
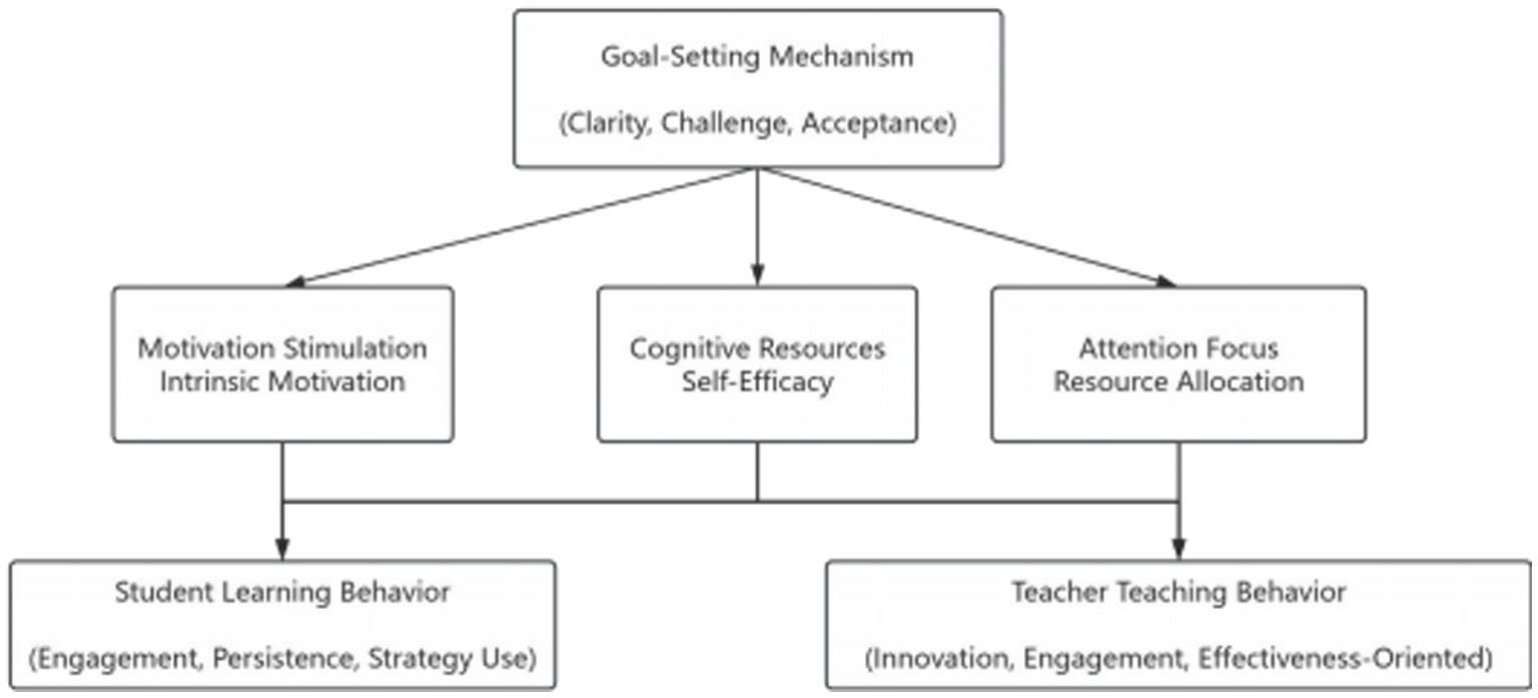
Hypothesized mediation and moderation model: Teacher Care (TC) positively affects Student Mental Well-being (MWB) directly and indirectly via Grit (perseverance) and Resilience (adversity coping). Student AI-interaction (SAI, engagement with AI educational tools) moderates the indirect paths. Solid lines = direct/mediated paths; dashed lines = moderation effects. *N*_students_ = 437.

The impact of goal-setting on behavior is not a direct linear relationship but operates through complex psychological mediating mechanisms, as illustrated in Figure 1. Motivation stimulation is the primary mediating pathway through which goal-setting affects behavior; clear and challenging goals can stimulate individual intrinsic motivation, generating strong desires to achieve goals. This motivation stimulation is reflected not only in behavioral initiation but more importantly in behavioral persistence and intensity. When individuals believe goals are meaningful and achievable, intrinsic motivation drives them to invest more time and energy, adopting more effective strategies to complete tasks.

Self-efficacy, as another important psychological mediating variable, reflects the degree of individual belief in their ability to successfully complete specific tasks. Goal-setting helps enhance individual self-efficacy by providing clear success standards and achievement pathways. Individuals with high self-efficacy demonstrate stronger persistence when facing difficulties, are more willing to choose challenging tasks, and show better self-regulation abilities during execution. Conversely, low self-efficacy may lead individuals to avoid challenges, reduce effort levels, or give up prematurely.

Attention concentration and cognitive resource allocation constitute the cognitive mediating mechanisms through which goal-setting affects behavior. Clear goals help individuals focus limited cognitive resources on relevant tasks, reducing interference from irrelevant information. This attention focusing effect not only improves task execution efficiency but also enhances the depth of learning and work. Meanwhile, the existence of goals provides individuals with reference standards for evaluating progress and adjusting strategies, making cognitive resource allocation more reasonable and effective (see Figure 1).

### 2.4 Research hypotheses and theoretical model construction

Based on the integrated analysis of goal-setting theory and self-determination theory, combined with the specific contextual characteristics of educational management practice, this study constructed a comprehensive theoretical model of how goal-setting mechanisms influence student and teacher behavior. Given the theoretical predictions derived from established frameworks, we propose the following hypotheses tested using two-tailed significance tests to maintain statistical rigor:

*Student behavior hypotheses*: H1a—Goal-setting mechanisms are positively associated with student learning behavior. H1b—Learning motivation mediates the relationship between goal-setting mechanisms and student learning behavior, such that goal-setting mechanisms enhance learning motivation, which in turn promotes student learning behavior. H1c—Self-efficacy mediates the relationship between goal-setting mechanisms and student learning behavior, such that goal-setting mechanisms enhance self-efficacy, which in turn promotes student learning behavior. H1d—Learning motivation and self-efficacy play a chain mediating role in the relationship between goal-setting mechanisms and student learning behavior, such that goal-setting mechanisms enhance learning motivation, which subsequently increases self-efficacy, ultimately promoting student learning behavior.
*Teacher behavior hypotheses*: H2a—Goal-setting mechanisms are positively associated with teacher teaching behavior. H2b—Professional identity mediates the relationship between goal-setting mechanisms and teacher teaching behavior, such that goal-setting mechanisms enhance professional identity, which in turn promotes teacher teaching behavior. H2c—Teaching efficacy mediates the relationship between goal-setting mechanisms and teacher teaching behavior, such that goal-setting mechanisms enhance teaching efficacy, which in turn promotes teacher teaching behavior. H2d—Professional identity and teaching efficacy play a chain mediating role in the relationship between goal-setting mechanisms and teacher teaching behavior, such that goal-setting mechanisms enhance professional identity, which subsequently increases teaching efficacy, ultimately promoting teacher teaching behavior.
*Moderation effect hypotheses*: H3a—Organizational support moderates the relationship between goal-setting mechanisms and learning motivation, such that this relationship is stronger when organizational support is higher. H3b—Organizational support moderates the relationship between goal-setting mechanisms and self-efficacy, such that this relationship is stronger when organizational support is higher. H3c—Organizational support moderates the relationship between goal-setting mechanisms and professional identity, such that this relationship is stronger when organizational support is higher. H3d—Organizational support moderates the relationship between goal-setting mechanisms and teaching efficacy, such that this relationship is stronger when organizational support is higher. H3e—Organizational support moderates the direct relationship between goal-setting mechanisms and student learning behavior, such that this relationship is stronger when organizational support is higher. H3f—Organizational support moderates the direct relationship between goal-setting mechanisms and teacher teaching behavior, such that this relationship is stronger when organizational support is higher.

Student Behavior Model:


SB=α1+β1(GS)+β2(LM)+β3(SE)+β4(GS×OS)+ε1

Learning Motivation Mediation:


LM=α2+γ1(GS)+γ2(GS×OS)+ε2

Self-Efficacy Mediation:


SE=α3+γ3(GS)+γ4(LM)+γ5(GS×OS)+ε3

Teacher Behavior Model:


TB=α4+β5(GS)+β6(PI)+β7(TE)+β8(GS×OS)+ε4

Professional Identity Mediation:


PI=α5+γ6(GS)+γ7(GS×OS)+ε5

Teaching Efficacy Mediation:


TE=α6+γ8(GS)+γ9(PI)+γ10(GS×OS)+ε6

Variable descriptions:

Dependent variables: SB = Student Behavior, TB = Teacher Behavior.
Independent variable: GS = Goal-Setting Mechanism.
Mediating variables: LM = Learning Motivation, SE = Self-Efficacy, PI = Professional Identity, TE = Teaching Efficacy.
Moderating variable: OS = Organizational Support.
Coefficients: *α* = Intercept, *β* = Direct Effect Coefficient, *γ* = Mediation Path Coefficient, *ε* = Error Term.

Based on theoretical analysis and empirical research needs, the theoretical model constructed in this study reflects the complexity and multi-level nature of goal-setting mechanisms. The model not only considers direct effects but more importantly reveals the pathways of psychological mediating mechanisms, providing a theoretical framework for in-depth understanding of how goal-setting works in educational management. Meanwhile, the introduction of moderating variables in the model makes the theoretical framework more closely aligned with the actual context of educational management, laying a solid theoretical foundation for subsequent empirical testing.

## 3 Research design and empirical methods

This study employed a cross-sectional survey design examining psychological mechanisms through which goal-setting influences behavior. Data from 1,247 students and 358 teachers across 15 schools were analyzed using structural equation modeling for separate student/teacher samples, multilevel regression for school-level clustering, and bootstrapping for mediation testing.

### 3.1 Research hypotheses and conceptual model design

This study employed a cross-sectional survey design to examine the psychological mechanisms through which goal-setting mechanisms influence behavioral outcomes in educational settings. The cross-sectional approach was selected for several methodological and practical reasons. First, it enables efficient collection of data from large, geographically dispersed samples, which is essential for testing complex structural equation models requiring substantial statistical power. Second, cross-sectional designs are appropriate for initial testing of theoretically-derived mediation and moderation models, establishing patterns of associations that warrant subsequent longitudinal investigation. Third, the design allows simultaneous measurement of all constructs within a consistent temporal frame, reducing potential confounds introduced by historical events or developmental changes that might occur across extended data collection periods. The study design incorporated several features to enhance internal validity and generalizability. A dual-population approach simultaneously examined students (*N* = 1,247) and teachers (*N* = 358) to test whether goal-setting mechanisms operate through similar or distinct psychological pathways across different educational roles. This parallel-groups design permits comparative analysis of mediating processes while acknowledging that learners and instructional professionals possess fundamentally different motivational structures and organizational positions. Multi-site sampling across 15 schools spanning three geographical regions (eastern, central, and western China) and three educational levels (junior high, senior high, university) was implemented to enhance ecological validity and sample heterogeneity, enabling preliminary examination of model generalizability across diverse educational contexts. Data collection employed standardized survey protocols to maximize measurement consistency. Student surveys were administered in group settings during non-instructional time under supervision of trained research assistants who followed detailed administration scripts. Teacher surveys utilized both online and offline modalities with unique access codes to accommodate diverse scheduling constraints while maintaining response confidentiality. All surveys were anonymous, with participants identified only by randomly-assigned identification numbers. The survey battery required approximately 25–30 min for students and 30–35 min for teachers to complete, designed to balance comprehensive construct measurement with minimization of respondent fatigue. To address common limitations of cross-sectional survey research, several methodological safeguards were implemented. Temporal separation was introduced by administering predictor and outcome measures in counterbalanced order, with half of participants completing goal-setting and organizational support items first, and half completing behavioral outcome items first, to reduce potential priming effects. Common method bias was assessed through multiple statistical techniques including Harman’s single-factor test and confirmatory factor analysis comparing single-factor versus hypothesized multi-factor measurement models. Social desirability response bias was minimized through emphasis on anonymity, inclusion of reverse-coded items, and explicit instructions emphasizing that there were no “right” or “wrong” answers. Non-response bias was evaluated by comparing early versus late respondents on key demographic and substantive variables. The design acknowledges important limitations inherent to cross-sectional data. Most critically, the design precludes definitive causal inferences; while the theoretical model proposes that goal-setting influences behavior through psychological mediators, alternative directional relationships (e.g., behavior influencing motivation, which subsequently shapes goal perceptions) cannot be ruled out with cross-sectional data. The absence of temporal precedence means that observed associations, while consistent with theoretical predictions, require corroboration through longitudinal or experimental designs to establish causality. Additionally, unmeasured third variables (e.g., prior academic achievement, personality traits, school climate factors not captured in our organizational support measure) may account for observed relationships. These limitations are addressed explicitly in the Discussion section, with concrete recommendations for future longitudinal and experimental research to establish causal mechanisms definitively. Despite these limitations, cross-sectional designs remain valuable for theory testing when: (1) strong theoretical predictions exist regarding expected patterns of associations; (2) temporal dynamics are not central to theoretical predictions (i.e., theories predict relationships rather than sequences); (3) practical or ethical constraints preclude experimental manipulation; and (4) findings are interpreted appropriately as patterns of covariation rather than established causal effects. Our study meets these criteria, employing cross-sectional data to test theoretically-derived predictions about psychological mediation and contextual moderation of goal-setting effects, with full acknowledgment of the need for complementary longitudinal research to establish temporal dynamics and directionality conclusively.

### 3.2 Variable measurement and questionnaire development

The construction of the variable measurement system adopted a strategy combining mature, validated scales with contextualized adaptations for Chinese educational settings, ensuring both scientific validity and cultural appropriateness. Rather than developing entirely new instruments, we adapted well-established scales from the international literature, subjecting them to rigorous translation and validation procedures. All English-language scales underwent translation-back-translation processes conducted by independent bilingual researchers with expertise in educational psychology, with any discrepancies resolved through expert panel discussions. Following translation, cognitive pretesting was conducted with 30 students and 15 teachers to ensure item comprehension, cultural relevance, and face validity within the Chinese educational context.

Goal-setting mechanism measurement adapted Locke and Latham’s classic four-dimensional framework into 16 items covering goal clarity (4 items; e.g., “My goals are clearly defined and specific”), challenge (4 items; e.g., “Goals set for me are appropriately challenging”), acceptance (4 items; e.g., “I fully accept established goals”), and feedback (4 items; e.g., “I regularly receive progress feedback”). All items used 7-point Likert scales (1 = strongly disagree, 7 = strongly agree). Confirmatory factor analysis during pretesting demonstrated acceptable fit: *χ*^2^/df = 2.18, CFI = 0.94, RMSEA = 0.058; factor loadings ranged from 0.68 to 0.89; composite reliability (CR) = 0.92; average variance extracted (AVE) = 0.65.

Student learning behavior measurement adapted three dimensions from Pintrich et al.’s widely-validated Motivated Strategies for Learning Questionnaire (MSLQ) (Yuntao, 2024), with modifications to reflect Chinese classroom contexts. The 12-item scale measured engagement (4 items; e.g., “I actively participate in class discussions”), persistence (4 items; e.g., “I persist through difficult assignments”), and strategy use (4 items; e.g., “I use effective organization strategies”). Pretest CFA yielded: *χ*^2^/df = 2.05, CFI = 0.95, RMSEA = 0.054; factor loadings 0.71–0.87; CR = 0.90; AVE = 0.62.

Teacher teaching behavior was measured using a 14-item scale assessing innovation (5 items; e.g., “I actively try new teaching methods”), engagement (5 items; e.g., “I invest substantial preparation time”), and effectiveness orientation (4 items; e.g., “I adjust strategies to improve outcomes”). Pretest CFA: χ^2^/df = 2.12, CFI = 0.96, RMSEA = 0.056; loadings 0.69–0.88; CR = 0.91; AVE = 0.64.

Learning motivation was measured using a 10-item two-dimensional scale adapted from the validated Academic Motivation Scale, covering intrinsic motivation (5 items) and extrinsic motivation (5 items). Self-efficacy adapted Bandura’s general self-efficacy scale into 8 items specific to learning contexts (e.g., “I am confident mastering difficult materials”). Professional identity was measured using a 12-item scale covering value identification, emotional attachment, and behavioral tendency dimensions. Teaching efficacy adapted the widely-used scale developed by Tschannen-Moran et al., covering instructional strategy efficacy, classroom management efficacy, and student engagement efficacy across 15 items.

**Figure 2:**
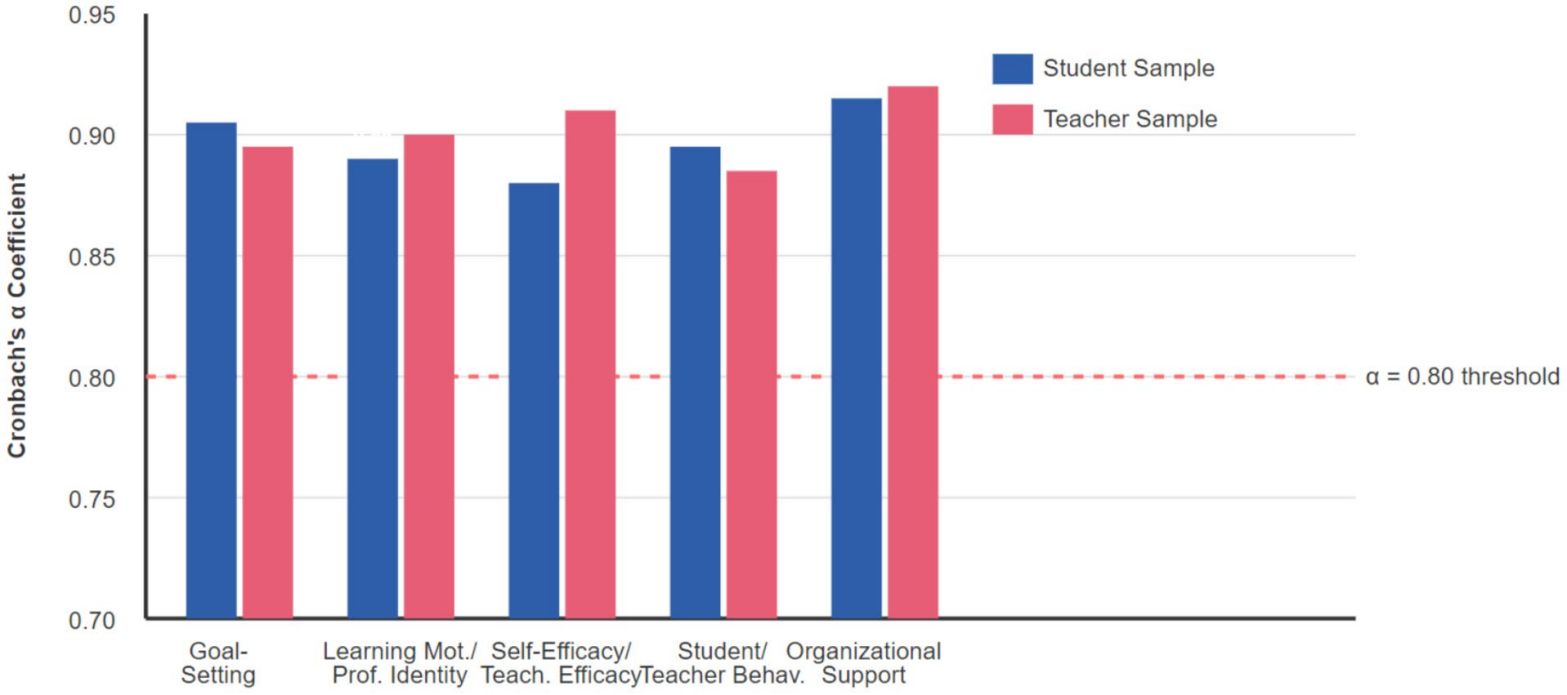
Internal consistency reliability (Cronbach’s *α*) for all measurement scales. All coefficients exceed 0.80 threshold. Detailed psychometric properties including CR and AVE reported in Table 2.

Organizational support measured three dimensions across 18 items: institutional support (6 items; e.g., “Clear policies support goal achievement”), resource support (6 items; e.g., “Adequate professional development resources”), and cultural support (6 items; e.g., “Culture encourages continuous improvement”), all rated on 7-point scales. Complete measurement scales with all items in both English and Chinese, detailed factor loadings, and comprehensive psychometric properties are provided in Supplementary Table S1. All scales demonstrated adequate reliability (Cronbach’s *α*: 0.86–0.93; CR: 0.87–0.94) and convergent validity (AVE: 0.61–0.68) in full sample analyses (Ifeanyieze et al., 2023) (see Figure 2). Complete measurement scales with item-level descriptive statistics, confirmatory factor analysis results (standardized factor loadings, modification indices, residual correlations), and additional psychometric evidence are provided in Supplementary Tables S1–S4. Correlation matrices separated by educational stage and region are available in Supplementary Tables S5–S7.

### 3.3 Sample selection and data collection procedures

Data collection procedures strictly adhered to research ethics requirements and scientific standards. This study received ethical approval from the Institutional Review Board of Changchun Humanities and Sciences College (approval number: CHSC-2025-014) prior to any data collection activities. The approval process included comprehensive review of research procedures, consent protocols, data protection measures, and potential risks to participants. Written informed consent was obtained from parents or legal guardians of all minor students (under 18 years old), with additional written assent obtained from the student participants themselves. Adult students (18 years and older) and all teacher participants provided direct written informed consent. All consent forms clearly explained the study’s purpose and procedures, participants’ rights to voluntary participation and withdrawal at any time without penalty, confidentiality protections and data security measures, potential benefits and minimal risks associated with participation, and contact information for questions or concerns.

Sample selection adopted a two-stage stratified-convenience sampling approach to ensure representation across diverse educational contexts while maintaining feasibility. In the first stage, stratified sampling selected five schools from each of three major geographical regions (eastern, central, and western China), stratified by educational level (junior high school, senior high school, university). Selection criteria for participating schools included: moderate institutional size (1,000–3,000 students), average-or-above educational quality indicators, complete management systems and goal-setting practices, and willingness to cooperate with research activities. In the second stage, convenience sampling invited all students from randomly selected classes within each school and all available teachers to participate. To assess potential non-response bias, we compared early respondents (first 50% of returned surveys) with late respondents (last 50%) on key demographic variables, finding no significant differences (all *p* > 0.10), suggesting non-response bias was minimal. Additionally, participating schools’ performance indicators did not differ significantly from provincial averages (independent samples *t*-test, *p* = 0.34), supporting sample representativeness (Ifeanyieze et al., 2023).

In the preliminary stage, contact was established with academic affairs departments and teacher development centers of each school to obtain institutional research permission and support. Student questionnaire surveys were conducted through class-based group administration during non-instructional time, with trained research assistants following standardized protocols. Teacher surveys combined online and offline methods with unique access codes. The entire data collection process lasted 3 months (September–November 2024), ensuring data timeliness and consistency (Wensheng and Hao, 2022). Quality control included pilot testing, on-site supervision, completeness checks, and double-entry verification. Finally, 1,247 valid student questionnaires (91.3% response rate) and 358 valid teacher questionnaires (89.7% response rate) were collected with balanced regional and educational-level distribution (see Table 1).

**Table 1:** Sample distribution and basic characteristics.

Characteristics	Category	Frequency	%
Student sample (*N* = 1,247)			
Gender	Male	612	49.1
	Female	635	50.9
Age	13–15 years	418	33.5
	16–18 years	426	34.2
	19–21 years	327	26.2
	22+ years	76	6.1
	M ± SD	17.2 ± 2.8	–
Educational stage	Junior high	418	33.5
	Senior high	426	34.2
	University	403	32.3
Region	Eastern	415	33.3
	Central	416	33.4
	Western	416	33.3
Teacher sample (*N* = 358)			
Gender	Male	142	39.7
	Female	216	60.3
Teaching experience	1–5 years	89	24.9
	6–10 years	98	27.4
	11–20 years	112	31.3
	21+ years	59	16.5
	M ± SD	11.6 ± 7.3	—
Education level	Bachelor’s	156	43.6
	Master’s	187	52.2
	Doctoral	15	4.2
Educational stage	Junior high	119	33.2
	Senior high	125	34.9

Response rates: students 91.3%, teachers 89.7%. Regional distribution balanced per stratified design.

### 3.4 Data analysis methods and model testing strategies

Data analysis employed multi-level statistical methods using Mplus 8.5 for SEM and SPSS 26.0 for preliminary analyses. Descriptive statistics calculated means, standard deviations, skewness, and kurtosis. Given large samples, normality was evaluated through skewness/kurtosis values (±2 range) rather than Kolmogorov–Smirnov tests, which are overly sensitive. Correlation analysis used Pearson coefficients; variance inflation factors (VIF) tested multicollinearity.

Structural equation modeling employed maximum likelihood estimation with robust standard errors (MLR) for potential non-normality. Missing data (students 3.2%, teachers 2.8%) were handled via full information maximum likelihood (FIML) under missing-at-random assumptions. Measurement models evaluated reliability and validity through CR, AVE, and factor loadings. Fit indices: *χ*^2^/df < 3, CFI > 0.95, TLI > 0.95, RMSEA<0.06, SRMR<0.08.

Participants nested within schools necessitated multilevel analyses. Intraclass correlations (ICC = 0.08–0.12) indicated 8–12% school-level variance. Multilevel path models included school clustering with random intercepts, though primary effects remained consistent with single-level models. Common method bias was assessed via Harman’s single-factor test (first factor = 28.4%, below 50% threshold) and CFA marker-variable approach (ΔCFI = 0.008), indicating CMB was not a major concern.

Mediation testing used bias-corrected bootstrap with 5,000 resamples generating 95% confidence intervals for indirect effects. Chain mediation pathways were tested separately (e.g., GS → LM → SB; GS → SE → SB; GS → LM → SE → SB), calculating total indirect effects while controlling demographics. Moderation used interaction term regression with mean-centered variables. Simple slope analysis examined effects at low (−1 SD), mean, and high (+1 SD) moderator levels, with Johnson-Neyman analyses identifying significance regions. Interaction plots visualized moderation patterns.

Model testing reflected rigor and systematicity. Competing models (full mediation, partial mediation, direct effect) were compared using AIC and BIC. Cross-validation randomly divided samples into training and validation sets. Sensitivity analysis tested robustness through extreme value deletion. Multi-group analysis tested universality across educational stages and regions using chi-square difference tests.

## 4 Empirical results and statistical analysis

### 4.1 Descriptive statistics and correlation analysis

Statistical analysis results showed that all research variables exhibited good normal distribution characteristics, with skewness and kurtosis values within acceptable ranges (between −2 and +2), laying a foundation for subsequent parametric statistical testing. Goal-setting mechanisms had a mean of 5.23 (SD = 1. 14) in the student sample and 5.41 (SD = 1.08) in the teacher sample, indicating that overall, goal-setting mechanisms in schools were at a moderate-to-high level. Student learning behavior had a mean of 4.89 (SD = 1.26), and teacher teaching behavior had a mean of 5. 12 (SD = 1. 15), reflecting some room for improvement in behavioral performance among research subjects. Among mediating variables, learning motivation had a mean of 4.76 (SD = 1.33), self-efficacy had a mean of 4.93 (SD = 1. 19), professional identity had a mean of 5.28 (SD = 1.02), and teaching efficacy had a mean of 5.15 (SD = 1.07), results that were basically consistent with theoretical expectations (Wensheng and Hao, 2022).

Correlation analysis revealed significant positive association patterns among variables, providing preliminary support for hypothesis testing. Goal-setting mechanisms showed moderate positive correlation with student learning behavior (*r* = 0.58, *p* < 0.001) and significant positive correlation with teacher teaching behavior (*r* = 0.52, *p* < 0.001). Correlation analysis results for mediating variables further validated the reasonableness of the theoretical model, with learning motivation showing strong positive correlation with self-efficacy (*r* = 0.64, *p* < 0.001), and professional identity showing significant positive correlation with teaching efficacy (*r* = 0.61, *p* < 0.001). Organizational support, as a moderating variable, showed moderate positive correlations with all core variables, with correlation coefficients ranging from 0.35 to 0.48, indicating that organizational support plays an important contextual role in goal-setting processes (Michael et al., 2023). Variance inflation factor test results showed that VIF values for all variables were less than 3.0, ruling out the existence of serious multicollinearity problems (see Figure 2). Given all measures relied on self-report questionnaires, common method bias was assessed using Harman’s single-factor test, which showed the first unrotated factor explained 28.4% of variance (below 50% threshold), and CFA comparison between hypothesized multi-factor models versus single-factor models showed superior fit for multi-factor structure (Δ*χ*^2^ = 6,609.25, Δdf = 25, *p* < 0.001), indicating distinct constructs were measured and CMB was unlikely to substantially distort relationships (see Table 2).

**Table 2:** Descriptive statistics, reliability, and correlation matrices.

Part A: descriptive statistics and reliability indices									
Variable	Sample	*N*	Mean	SD	Skew	Kurt	α	CR	AVE
Goal-setting	Student	1,247	5.23	1.14	−0.32	−0.18	0.91	0.92	0.65
	Teacher	358	5.41	1.08	−0.28	−0.15	0.89	0.90	0.63
Learning motivation	Student	1,247	4.76	1.33	−0.15	−0.42	0.88	0.89	0.62
Self-efficacy	Student	1,247	4.93	1.19	−0.21	−0.35	0.86	0.87	0.61
Student behavior	Student	1,247	4.89	1.26	−0.18	−0.29	0.89	0.90	0.64
Professional identity	Teacher	358	5.28	1.02	−0.35	0.12	0.90	0.91	0.66
Teaching efficacy	Teacher	358	5.15	1.07	−0.31	0.08	0.92	0.93	0.68
Teacher behavior	Teacher	358	5.12	1.15	−0.24	−0.16	0.87	0.88	0.62
Org. support	Student	1,247	4.68	1.28	−0.12	−0.45	0.93	0.94	0.67
	Teacher	358	4.85	1.21	−0.19	−0.38	0.94	0.94	0.68

Variable	1	2	3	4	5
Part B: student sample correlations (*N* = 1,247)					
1. Goal-setting	–				
2. Learning motivation	0.48***	–			
3. Self-efficacy	0.51***	0.64***	–		
4. Student behavior	0.58***	0.71***	0.66***	–	
5. Org. support	0.42***	0.38***	0.35***	0.41***	–
Part C: teacher sample correlations (*N* = 358)					
1. Goal-setting	–				
2. Professional identity	0.44***	–			
3. Teaching efficacy	0.46***	0.61***	–		
4. Teacher behavior	0.52***	0.67***	0.69***	–	
5. Org. support	0.43***	0.45***	0.48***	0.48***	–

α = Cronbach’s alpha; CR = Composite Reliability; AVE = Average Variance Extracted. All skewness/kurtosis within ±2. ***p < 0.001. VIF<3.0 for all variables. Missing data handled via FIML.

### 4.2 Testing the impact of goal-setting mechanisms on student behavior

The structural equation modeling analysis results confirmed the significant positive impact of goal-setting mechanisms on student learning behavior, validating the expected hypotheses of the theoretical model. The model fit indices all reached good standards, with *χ*^2^/df = 2.34, CFI = 0.96, TLI = 0.95, RMSEA = 0.052, SRMR = 0.048.indicating good fit between the theoretical model and actual data (Junqi, 2023). Path analysis results showed that goal-setting mechanisms had a significant direct positive association with student learning behavior (*β* = 0.42, SE = 0.05, *t* = 8.76, *p* < 0.001), supporting hypothesis H1a. Control variables showed gender weakly predicted behavior (*β* = 0.08, SE = 0.03, *p* < 0.05, females higher), age was non-significant (*β* = 0.04, *p* > 0.05), and senior high students showed stronger goal-orientation (*β* = 0.12, SE = 0.04, *p* < 0.01). Multi-group analysis confirmed path coefficients did not differ across regions (Δ*χ*^2^ = 3.42, df = 2, *p* > 0.05), validating cross-regional stability. Goal-setting mechanisms explained 18% of student learning behavior variance, reflecting goal-setting’s important role in promoting positive student behavior.

Dimensional analysis further revealed the differentiated impact patterns of various components of goal-setting mechanisms on student behavior. Goal clarity had the most significant impact on student learning behavior (*β* = 0.38, *p* < 0.001), reflecting that clear and specific learning goals can provide students with clear directions for effort and evaluation standards. The impact coefficient of goal challenge was 0.31 (*p* < 0.001), indicating that moderately challenging goals can stimulate students’ learning potential and intrinsic motivation. The impact of goal acceptance was relatively small but still significant (*β* = 0.26, *p* < 0.01), showing that students’ degree of identification with goals directly affects the intensity of their behavioral investment. The impact coefficient of feedback mechanisms was 0.29 (*p* < 0.001), validating the important value of timely and effective feedback in guiding student learning behavior adjustment.

**Figure 3:**
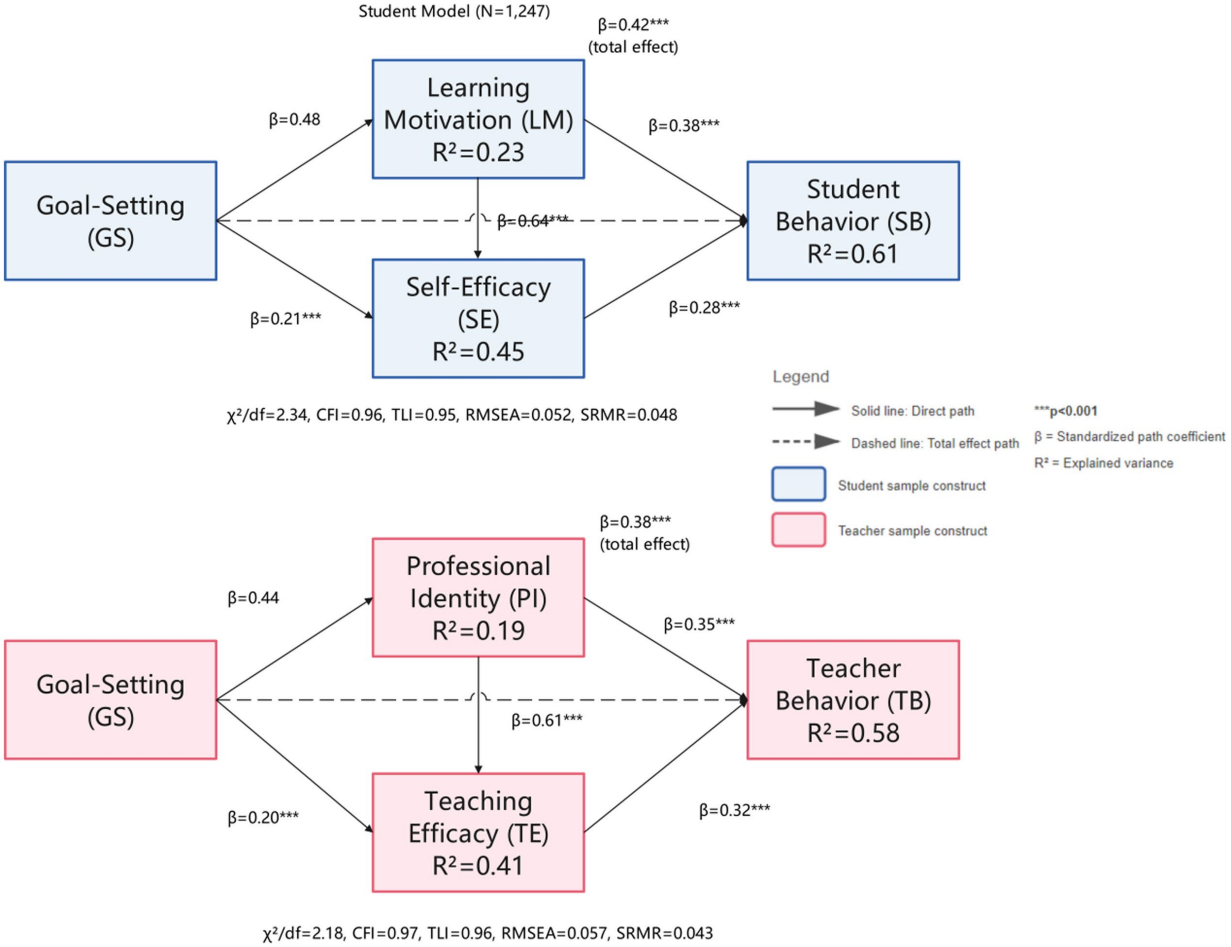
Structural model path coefficients. All paths significant at *p* < 0.001. Standardized coefficients (*β*) displayed on paths. *R*^2^ values indicate explained variance percentages. Student model: *N* = 1,247, *χ*^2^/df = 2.34, CFI = 0.96, TLI = 0.95, RMSEA = 0.052 (90%CI[0.047,0.058]), SRMR = 0.048. Teacher model: *N* = 358, *χ*^2^/df = 2.18, CFI = 0.97, TLI = 0.96, RMSEA = 0.057 (90%CI[0.049,0.065]), SRMR = 0.043. Control variables (gender, age/experience, education level) included but not shown for clarity.

Control variable analysis showed that individual characteristic factors played a certain role in the model. Gender had a weak but significant impact on student learning behavior (*β* = 0.08, *p* < 0.05), with female students performing slightly better than male students in learning behavior. Age had no significant impact (*β* = 0.04, *p* > 0.05), indicating that the effectiveness of goal-setting mechanisms was consistent across different age groups of students. Comparative analysis of educational stages found that high school students showed stronger goal-oriented behavior patterns compared to middle school and university students (*β* = 0. 12, *p* < 0.01), which may be closely related to the academic pressure and goal-oriented educational environment faced during high school. Multi-group analysis results showed that the impact of goal-setting mechanisms on student behavior did not differ significantly across different regions (Δχ^2^ = 3.42, *p* > 0.05), validating the cross-contextual stability of the theoretical model (see Figure 3).

### 4.3 Validation of goal-setting mechanisms’ impact on teacher behavior

The structural equation modeling analysis of the teacher group also achieved good fit effects, with all indicators reaching recommended standards: *χ*^2^/df = 2.18, CFI = 0.97, TLI = 0.96, RMSEA = 0.057 (90%CI[0.049,0.065]), SRMR = 0.043, confirming the effectiveness of the goal-setting-teacher behavior theoretical model. Path analysis results showed that goal-setting mechanisms had a significant positive impact on teacher teaching behavior (*β* = 0.38, *t* = 7.89, *p* < 0.001), validating the establishment of hypothesis H2a. This finding indicates that scientific goal-setting systems can effectively stimulate teachers’ teaching innovation motivation and improve teaching investment and effectiveness orientation. Compared to the student group, the teacher group showed stronger goal internalization tendencies, with goal-setting mechanisms able to explain 22% of the variance in teacher teaching behavior, reflecting the important value of goal-setting in promoting teacher professional development (Qiang, 2023).

Analysis of teacher behavior influence mechanisms revealed the moderating role of professional characteristics on goal-setting effectiveness. Teachers with different years of experience showed differentiated patterns in goal-setting response, with mid-level teachers (6–15 years of experience) showing the highest sensitivity to goal-setting (*β* = 0.43, *p* < 0.001). This group is in a critical period of professional development and has strong needs for clear development goals. Beginning teachers (1–5 years of experience) had relatively lower response coefficients (*β* = 0.31, *p* < 0.01), possibly related to their professional abilities still being in the formation stage. Senior teachers (16+ years of experience) had moderate response levels (*β* = 0.36, *p* < 0.001), indicating that experienced teachers, despite having mature teaching abilities, still need appropriate goal guidance to maintain professional development motivation (Lia and Silke, 2023).

Subject characteristic analysis showed certain differences in goal-setting responses among teachers of different subjects. Science teachers showed stronger goal-oriented behavior patterns compared to humanities teachers (*β* difference = 0.08, *p* < 0.05), possibly related to science teaching goals being relatively clearer and more specific. Vocational education teachers showed significantly higher goal response levels than basic education teachers (*β* = 0.42 vs. 0.35, *p* < 0.01), reflecting that vocational education’s clear employment orientation makes goal-setting more easily translated into specific teaching behaviors. Multi-group SEM with chi-square difference tests examined experience-based differences. Mid-career teachers (6–15 years) showed significantly stronger goal-setting→behavior associations (*β* = 0.43, SE = 0.05) versus novice teachers (1–5 years: *β* = 0.31, SE = 0.07; Δχ^2^ = 6.82, df = 1, *p* < 0.01) and senior teachers (16 + years: *β* = 0.36, SE = 0.06; Δχ^2^ = 4.15, df = 1, *p* < 0.05), suggesting mid-career represents peak goal sensitivity. Subject comparisons using interaction regression showed science teachers exhibited stronger goal-orientation than humanities teachers (*β*_interaction_ = 0.08, SE = 0.03, z = 2.67, *p* < 0.05). Educational background analysis found master’s degree holders showed strongest responsiveness (*β* = 0.41, SE = 0.05), followed by bachelor’s (*β* = 0.34, SE = 0.04) and doctoral holders (*β* = 0.29, SE = 0.08), though confidence intervals overlapped, requiring cautious interpretation. Analysis of teacher educational background found that teachers with master’s degrees showed the strongest goal responsiveness (*β* = 0.41, *p* < 0.001), followed by those with bachelor’s degrees (*β* = 0.34, *p* < 0.001), and those with doctoral degrees were relatively lower (*β* = 0.29, *p* < 0.01). This difference may be related to the career expectations and development needs of teachers at different educational levels.

### 4.4 Empirical analysis of psychological mediation and moderation effects

Mediation effect testing using 5,000 bootstrap resamples with bias-corrected 95% confidence intervals confirmed psychological variables’ mediating roles. Student analysis showed learning motivation significantly mediated goal-setting→behavior (indirect effect = 0.19, 95%CI[0.13,0.25]), validating H1b. Self-efficacy mediation was significant (indirect effect = 0.10, 95%CI[0.06,0.15]), supporting H1c. Learning motivation and self-efficacy formed a chain pathway (GS → LM → SE → SB: 0.08, 95%CI[0.05,0.12]), with total indirect effect = 0.21 (95%CI[0.15,0.28]), validating H1d. Controlling demographics, all effects remained significant with changes<0.02 (Eunyoung et al., 2023).

Teacher analysis showed professional identity mediated goal-setting→behavior (indirect effect = 0.18, 95%CI[0.11,0.24]), supporting H2b. Teaching efficacy mediation was significant (indirect effect = 0.11, 95%CI[0.07,0.16]), validating H2c. Chain mediation (GS → PI→TE → TB: 0.09, 95%CI[0.05,0.14]) yielded total indirect effect = 0.19 (95%CI[0.12,0.26]), confirming H2d. With covariates controlled, all effects remained robust. Effect decomposition revealed direct effects accounted for 66.7% of total effects in students (direct = 0.42, total = 0.63), while teachers showed 38.2% indirect proportion (indirect = 0.19, direct = 0.38, total = 0.57), indicating teacher behavior is more influenced by psychological cognitive factors (Yuheng et al., 2023).

Moderation analysis revealed organizational support’s contextual role. Interaction regression with mean-centered variables showed significant goal-setting × organizational support interactions for learning motivation (β_int = 0.15, SE = 0.04, *t* = 3.42, *p* < 0.001), supporting H3a, and self-efficacy (β_int = 0.12, SE = 0.04, *t* = 2.87, *p* < 0.01), validating H3b. Simple slope analysis indicated under high support (+1 SD), goal-setting→motivation association was stronger (β_high = 0.58, SE = 0.05, 95%CI[0.48,0.68]) versus low support (−1 SD: β_low = 0.38, SE = 0.05, 95%CI[0.28,0.48]; z = 2.83, *p* < 0.01). Teacher analyses showed support moderated goal-setting→professional identity (β_int = 0.18, SE = 0.04, *t* = 4.15, *p* < 0.001) and teaching efficacy (β_int = 0.14, SE = 0.04, *t* = 3.28, *p* < 0.01), supporting H3c-H3d. Johnson-Neyman analysis indicated goal-setting effects on professional identity became non-significant only when support fell below 2.8 (10th percentile), suggesting widespread benefit. Comparing R^2^ values, organizational support enhanced goal-setting effectiveness by 25–30% (ΔR^2^: 0.06–0.09), emphasizing organizational environment’s practical importance. Sensitivity analysis removing 10% extreme values yielded consistent results (all β_int significant, changes<0.03), validating robustness.

Effect size analysis further revealed the relative importance of various psychological mechanisms. In the student group, direct effects accounted for 66.7% of total effects, while indirect effects accounted for 33.3%, indicating that goal-setting influences behavior through both direct pathways and psychological mediation pathways. In the teacher group, the proportion of indirect effects was relatively higher, reaching 38.2%, reflecting that teacher behavior is more influenced by psychological cognitive factors. Analysis of moderation effects showed that organizational support could enhance the effectiveness of goal-setting by approximately 25–30%,emphasizing the important value of organizational environment in goal management practice. Sensitivity analysis through removing 10% of extreme values and re-testing showed that all major results remained stable, validating the robustness of the analysis results (see Table 3).

**Table 3:** Simple slopes at different organizational support levels.

Outcome	Sample	OS level	*β*	SE	95%CI	*t*
Learning motivation	Student	Low (−1 SD)	0.38	0.05	[0.28,0.48]	7.60***
		High (+1 SD)	0.58	0.05	[0.48,0.68]	11.60***
Self-efficacy	Student	Low (−1 SD)	0.41	0.05	[0.31,0.51]	8.20***
		High (+1 SD)	0.55	0.05	[0.45,0.65]	11.00***
Student behavior	Student	Low (−1 SD)	0.35	0.05	[0.25,0.45]	7.00***
		High (+1 SD)	0.49	0.05	[0.39,0.59]	9.80***
Professional identity	Teacher	Low (−1 SD)	0.32	0.06	[0.20,0.44]	5.33***
		High (+1 SD)	0.56	0.06	[0.44,0.68]	9.33***
Teaching efficacy	Teacher	Low (−1 SD)	0.36	0.06	[0.24,0.48]	6.00***
		High (+1 SD)	0.54	0.06	[0.42,0.66]	9.00***
Teacher behavior	Teacher	Low (−1 SD)	0.31	0.06	[0.19,0.43]	5.17***
		High (+1 SD)	0.45	0.06	[0.33,0.57]	7.50***

OS, Organizational support; *β* = standardized coefficient of goal-setting on outcome at specified level; Low = Mean – 1 SD; High = Mean + 1 SD. ***p < 0.001.

Additionally, organizational support significantly moderated the direct relationship between goal-setting and student behavior (β_int = 0.11, SE = 0.04, *t* = 2.65, *p* < 0.01), supporting H3e. Simple slope analysis showed that under high organizational support (+1 SD), the direct effect of goal-setting on student behavior was β_high = 0.49 (SE = 0.05, 95%CI[0.39,0.59]), compared to β_low = 0.35 (SE = 0.05, 95%CI[0.25,0.45]) under low support (−1 SD), with the difference being significant (z = 2.00, *p* < 0.05). Similarly, organizational support moderated the direct relationship between goal-setting and teacher behavior (β_int = 0.13, SE = 0.04, *t* = 3.05, *p* < 0.01), validating H3f. Under high support, the direct effect was β_high = 0.45 (SE = 0.06, 95%CI[0.33,0.57]) versus β_low = 0.31 (SE = 0.06, 95%CI[0.19,0.43]) under low support (*z* = 2.33, *p* < 0.05). These findings indicate that organizational support not only moderates the indirect pathways through psychological mediators but also strengthens the direct associations between goal-setting and behavioral outcomes.

## 5 Discussion and implications of research findings

### 5.1 Theoretical explanation of goal-setting psychological mechanisms and academic contributions

The empirical research results revealed complex psychological pathways through which goal-setting mechanisms operate in educational contexts, providing important theoretical contributions to deepening goal-setting theory application in education. The research found that goal-setting functions not simply as an external driving mechanism, but shows associations through stimulating intrinsic motivation, enhancing efficacy beliefs, and other deep psychological variables. This finding enriches Locke and Latham’s classic goal-setting theory by specifying internal mechanisms. Motivation mediation aligns with self-determination theory’s emphasis on intrinsic motivation, with goal-setting associated with sustained behavioral investment through satisfying autonomy and competence needs. Self-efficacy mediation validates efficacy beliefs’ key role in social cognitive theory, with clear goals providing ability assessment standards, influencing confidence judgments (Chen, 2024). Recent studies show similar patterns—Jaarsveld et al. (2025) found self-set goals predicted achievement through autonomous motivation, paralleling our LM → SE chain. However, our cross-sectional design precludes definitive causal inferences. While our model posits goal-setting influences behavior through psychological mediators, alternative directions (e.g., behavior influencing motivation, then shaping goal acceptance) cannot be ruled out. The associations observed are consistent with theoretical predictions, but longitudinal or experimental designs are necessary for establishing temporal precedence.

Chain mediation revealed psychological mechanisms’ progressive characteristics, with goal-setting promoting behavior through motivation stimulation and subsequent efficacy enhancement. This indicates educational goal management should focus not only on direct behavioral changes but also on gradual psychological construction. The LM → SE pathway in students and PI → TE pathway in teachers reflect cognitive evaluation and emotional experience’s important roles in goal internalization. Organizational support’s moderation mechanism extends goal-setting theory’s contextual boundaries, emphasizing organizational environment’s key value. Theoretical contributions also reflect educational contexts’ particularity, where goal-setting relies more on satisfying higher-order psychological needs like intrinsic motivation and professional identity compared to general organizational contexts.

### 5.2 In-depth analysis of differentiated impact on student and teacher behavior

The research found significant differentiated patterns in goal-setting response mechanisms between groups, reflecting essential differences in psychological need characteristics and professional development stages. Students’ goal responses mainly reflected motivation stimulation and efficacy enhancement, with mechanisms relying on immediate achievement experiences. Learning motivation, as main behavioral driver, shows strong plasticity and sensitivity in goal-setting, closely related to students being in capability development stages. Efficacy’s mediating role is relatively weaker, possibly related to immature self-cognition and uncertain ability judgments. Students’ goal acceptance is more influenced by external factors including teacher expectations, peer performance, and family support, thus showing strong contextual dependency.

Age differences showed relatively little impact on students’ goal responses, suggesting goal-setting mechanisms have universality across developmental stages. However, stronger goal-orientation among senior high students may reflect cohort effects (e.g., high-stakes examinations) rather than pure developmental effects. Disentangling cohort, period, and age effects requires longitudinal designs tracking individuals across time. Similarly, teachers’ experience significantly affected goal response, with mid-career teachers (6–15 years) showing strongest sensitivity. This could indicate genuine developmental trajectory where professional identity solidifies mid-career, or could reflect cohort effects where teachers entering 6–15 years ago experienced different training paradigms. Weak responses of beginning teachers may stem from professional identity not being fully established, while moderate responses of senior teachers may reflect relatively stable teaching patterns and habits. Cross-sequential designs would help distinguish developmental from cohort influences.

Teachers’ goal responses present obvious professional characteristics, with professional identity and teaching efficacy forming core pathways for behavioral change. Professional identity, unique to teachers, plays foundational roles in goal-setting, affecting not only acceptance but internalization depth and sustainability. Teachers’ goal responses show stronger value orientation, producing deeper identification and more lasting changes when goals align with professional values and philosophies. Teaching efficacy’s mediating role is more significant in teachers, reflecting professional workers’ self-ability judgments’ importance and professional confidence’s direct impact on teaching behavior.

### 5.3 Policy recommendations and application strategies for educational management practice

Based on empirical findings, educational management needs systematic goal-setting systems considering different subjects’ psychological characteristics and behavioral patterns. Recent research examining education policy implementation processes and influencing factors has emphasized the importance of evidence-based policy design and systematic execution strategies in educational management contexts (Junfeng et al., 2024). At policy levels, differentiated frameworks should be established, focusing on goal clarity and challenge design for students, stimulating learning motivation through specific, measurable goals and moderately challenging tasks. Student goal-setting should emphasize process feedback mechanisms, establishing timely progress monitoring and adjustment systems. For teachers, emphasis should be on goals’ value orientation and professional development content, combining personal goals with overall school strategies to enhance identification and internalization. Teacher goal-setting needs professional autonomy, allowing personalized development plans within frameworks while providing adequate support and growth platforms (Shakurova et al., 2022). Scholars have provided specific recommendations for implementing positive psychology interventions in school settings, emphasizing the importance of contextually-adapted implementation strategies and organizational support structures (Susan et al., 2020).

Specific recommendations include: (1) For students—implement semester-initial 2-h goal-setting workshops teaching SMART principles; establish bi-weekly 15-min individual check-ins with specific feedback; design tiered-difficulty assignments allowing appropriate challenge selection. (2) For teachers—allocate 40 h annually of protected professional development time for mid-career teachers (years 6–15); establish bi-weekly 90-min subject-based Professional Learning Communities; connect institutional goals with individual professional identity during annual reviews using reflective protocols. (3) Organizational support—establish transparent goal-setting procedures with quality rubrics; dedicate 5–7% of budgets to goal-support resources; launch annual Goal Achievement Forums where students/teachers share experiences.

Implementation timelines: Year 1 (planning, $5,000–10,000 for consulting); Year 2 (pilot in 20% of units, $15,000–25,000); Year 3 (scale to 70%, $25,000–40,000); Year 4 + (full implementation, $30,000–50,000 annually). These estimates suit medium-sized schools (1,500 students, 100 teachers), requiring proportional adjustment. Resource constraints may necessitate phased implementation prioritizing high-impact, low-cost strategies (feedback protocols, PLCs) before expensive technology investments. Practical application also requires establishing dynamic adjustment mechanisms, optimizing goal content and achievement pathways based on implementation effects and environmental changes.

### 5.4 Limitations and future research directions

However, the study has important limitations that must be acknowledged. First, the cross-sectional design precludes definitive causal inferences; while our findings align with theoretical predictions that goal-setting influences behavior through psychological mediators, alternative directional relationships (e.g., behavior influencing motivation, which then shapes goal acceptance) cannot be ruled out. Longitudinal designs tracking individuals across multiple time points are essential for establishing temporal precedence and testing potential reciprocal relationships among variables. Second, sample concentration in three Chinese regions limits generalizability to other cultural contexts and educational systems with different goal-setting practices and psychological orientations; cross-cultural replications are necessary to establish boundary conditions. Third, reliance exclusively on self-report measures introduces potential common method bias, although statistical tests (Harman’s single-factor test, CFA marker-variable technique) suggested this was not a major concern; nevertheless, future research should incorporate objective behavioral measures (e.g., actual academic performance, classroom observations) and multi-source ratings (e.g., teacher reports of student behavior, peer evaluations) to strengthen validity. Fourth, this study was not pre-registered, which limits transparency regarding potential analytical flexibility and hypothesis adjustments during analysis. While our hypotheses were theoretically derived *a priori*, lack of formal pre-registration means readers cannot verify that reported tests were planned rather than exploratory. Finally, our theoretical contribution lies in systematically integrating existing theories (goal-setting theory, self-determination theory, social cognitive theory) rather than proposing an entirely novel theoretical framework; while this integration advances understanding of psychological mechanisms in educational goal management, some may view this as incremental rather than transformative theoretical progress.

Future research directions include: (1) three-wave longitudinal panel designs with 6-month intervals to test temporal sequences and establish directionality of relationships; (2) randomized controlled trials experimentally manipulating goal-setting features (clarity, challenge, feedback) to provide definitive causal evidence; (3) systematic replications across Eastern and Western educational systems to clarify cultural boundary conditions and universality versus specificity of psychological mechanisms; (4) age-sequential designs to disentangle developmental (age effects), cohort (generational differences), and period (historical context) effects that our cross-sectional design cannot distinguish; and (5) multi-method studies combining self-reports with objective achievement data, behavioral observations, and physiological indicators of engagement to triangulate findings and reduce method bias.

## 6 Conclusion

Through constructing a goal-setting-behavior influence model based on psychological theory, this study revealed deep psychological mechanisms through which goal-setting mechanisms are associated with student and teacher behavior. Empirical analysis confirmed that goal-setting demonstrates not only direct associations with behavioral performance but more importantly shows relationships through psychological pathways such as intrinsic motivation and self-efficacy. In students, learning motivation and self-efficacy constitute the core mediation chain linking goal-setting with learning behavior; in teachers, professional identity and teaching efficacy play key mediating roles. This finding enriches goal-setting theory’s application in education and provides new theoretical perspectives for understanding individual behavior’s psychological driving mechanisms in educational management. At the practical level, results emphasize constructing personalized, supportive goal-setting systems, providing scientific basis for educational managers to optimize strategies. However, the study has important limitations. First, the cross-sectional design precludes causal inferences; while findings align with predictions that goal-setting influences behavior through psychological mediators, alternative directional relationships cannot be ruled out. Longitudinal designs tracking individuals across multiple time points are essential for establishing temporal precedence and testing reciprocal relationships. Second, sample concentration in three Chinese regions limits generalizability to other cultural contexts and educational systems, necessitating cross-cultural replications. Third, reliance on self-report measures introduces potential common method bias, though statistical tests suggested this was not a major concern; future research should incorporate objective behavioral measures and multi-source ratings. Future research directions include three-wave panel designs with 6-month intervals to test temporal sequences, randomized controlled trials manipulating goal-setting features to provide causal evidence, systematic replications across Eastern and Western educational systems to clarify cultural boundary conditions, and age-sequential designs to disentangle age, cohort, and period effects. Despite these limitations, this study provides robust cross-sectional evidence for psychological mediation mechanisms in educational goal-setting, offering evidence-based guidance for educational management practice while charting directions for future definitive causal research.

## Data Availability

The original contributions presented in the study are included in the article/Supplementary material, further inquiries can be directed to the corresponding author.

## References

[ref1] Ashraf A. (2022). Investigating sustainable education and positive psychology interventions in schools towards achievement of sustainable happiness and wellbeing for 21st century pedagogy and curriculum. Electrochem. Soc. Trans. 107. doi: 10.1149/10701.19481ECST

[ref2] Chen M. (2024). Study on the path of enhancing the effectiveness of college students’ education and management in the context of informatization. Appl. Math. Nonlinear Sci. 9, 607–612. doi: 10.2478/AMNS-2024-0395

[ref3] Dehtjare J. Uzule K. (2023). Sustainable higher education management: career drivers of academic staff. J. Teach. Educ. Sustain. 25, 89–105. doi: 10.2478/JTES-2023-0018

[ref4] Eunyoung K. Julie C. Foster E. R. (2023). Implementation strategies for occupational therapists to advance goal setting and goal management. Front. Health Serv. 3:1042029. doi: 10.3389/FRHS.2023.1042029 37351362 PMC10282647

[ref5] Guo K. Zhang J. Ansari A. W. H. (2025). Teacher care and mental wellbeing: exploring the role of grit, resilience, and AI-interaction in education management. Acta Psychol. 261:105977. doi: 10.1016/J.ACTPSY.2025.105977, 41289920

[ref6] Ifeanyieze F. O. Ede K. R. Ejiofor T. E. Onah O. Isiwu E. C. Nwankwo C. U. . (2023). Psychological intervention for career self-esteem among students of agricultural education programme. Medicine 102:e33886. doi: 10.1097/MD.0000000000033886 37233411 PMC10219719

[ref7] Jaarsveld V. M. G. Wong J. Baars M. Specht M. Paas F. (2025). Goal setting in higher education: how, why, and when are students prompted to set goals? A systematic review. Front. Educ. 9:1511605. doi: 10.3389/FEDUC.2024.1511605

[ref8] Jing X. (2024). Exploration of an innovative model of higher education management and student training mechanism based on cognitive mapping. Appl. Math. Nonlinear Sci. 9. doi: 10.2478/AMNS.2023.2.01548

[ref9] Junfeng D. Meifang L. Xibin H. (2024). A review of the EU’s digital education policy: implementation process and influencing factors. Modern Educ. Technol. 34, 113–122.

[ref10] Junqi L. A study on teacher time conflicts and management in after-school extended services in primary schools [D]. Nanjing Normal University, Nanjing, China 2023.

[ref11] Li X. Guo N. (2024). Analysis of factors influencing the accumulation of psychological capital of college students under the theory of cognitive psychology. Appl. Math. Nonlinear Sci. 9. doi: 10.2478/AMNS.2023.1.00268, 40909103

[ref12] Lia O. Silke H. (2023). Choosing connection: relational values as a career choice motivation predict teachers' relational goal setting. Front. Psychol. 14:1147276. doi: 10.3389/FPSYG.2023.1147276 37265960 PMC10230071

[ref13] Liu P. Yu H. (2025). The exploration of integrated learning algorithms in the comprehensive evaluation of college students’ mental health and the optimization of education management. Int. J. High Speed Elect. Syst. doi: 10.1142/S012915642540525X, 40951326

[ref14] Mahere M. S. (2025). Contributions of higher education reforms to sustainable development goals: some examples and experiences from the Department of Educational Administration and Leadership, Faculty of Education, University of Zimbabwe. J. Adult Contin. Educ. 31, 244–264. doi: 10.1177/14779714241261063

[ref15] Michael E. Nick K. Dat H. Roshini J. (2023). Improved trial success using a novel patient app for goal setting and digital education resources. Neuromodulation Technol. Neural Interface 26, S73–S73. doi: 10.1016/J.NEUROM.2023.04.125

[ref16] Qiang S. (2023). Combining humanized education management with psychological education on the intervention of depression in students. CNS Spectr. 28, S48–S49. doi: 10.1017/S1092852923003735

[ref17] Serra-Garcia M. Hansen T. K. Gneezy U. (2020). Can short psychological interventions affect educational performance? Revisiting the effect of self-affirmation interventions. Psychol. Sci. 31, 865–872. doi: 10.1177/0956797620923587, 32609078

[ref18] Shakurova V. M. Pashkevich V. V. Arakelyan R. A. . (2022). Target settings for modeling career guidance in the education system of the region. Sci. Educ. Today 12. doi: 10.15293/2658-6762.2205.06

[ref19] Susan M. Rachel B. Cotton K. B. (2020). Recommendations for positive psychology interventions in school settings. J. Posit. Psychol. 15, 653–656. doi: 10.1080/17439760.2020.1789709

[ref20] Wensheng W. Hao C. (2022). Educational reward and punishment and the effect of psychological intervention on adolescent depression. J. Environ. Public Health 2022:3919519. doi: 10.1155/2022/3919519, 36111067 PMC9470316

[ref21] Wynarczuk K. D. Julia S. Theresa S. (2025). Commentary on: “Collaborative goal-setting approaches to support participation of children with special educational needs”. Pediatr. Phys. Ther. 37, 345–345. doi: 10.1097/PEP.0000000000001215 40587598

[ref22] Yuheng L. Mladen R. Wei D. Jionghao L. Hassan K. Kirsten G. . (2023). Are deeper reflectors better goal-setters? AI-empowered analytics of reflective writing in pharmaceutical education. Comput. Educ. Artif. Intell. 5. doi: 10.1016/J.CAEAI.2023.100157

[ref23] Yuntao G. (2024). Application of positive psychological intervention in enhancing the effectiveness of mental health and ideological and political education in colleges and universities [J]. Chin. J. School Health 45, 1060–1217.

