# Supplementary material for: Goal-setting mechanisms in educational management: a psychological perspective on student and teacher behavior
Source: Front Psychol. 2026 Jan 30;16:1680752. doi: 10.3389/fpsyg.2025.1680752 (PMC12901404; doi:10.3389/fpsyg.2025.1680752)
Supplement: Supplementary file 1 [file Supplementary_file_1.docx]

**Table S1. Complete Measurement Scales: Items, Factor Loadings, and Psychometric Properties**

**Part A: Goal-Setting Mechanism Scale (16 items)**

**Dimension 1: Goal Clarity (4 items)**

| Item | English Version | Chinese Version | Loading | M | SD |
| --- | --- | --- | --- | --- | --- |
| GS1 | My learning/teaching goals are clearly defined and specific | 我的学习/教学目标清晰明确且具体 | 0.84 | 5.31 | 1.18 |
| GS2 | I know exactly what I need to accomplish in my studies/teaching | 我清楚地知道在学习/教学中需要完成什么 | 0.87 | 5.28 | 1.22 |
| GS3 | The expectations for my performance are well-articulated | 对我表现的期望阐述得很清楚 | 0.82 | 5.19 | 1.27 |
| GS4 | I have a clear understanding of the standards I need to meet | 我清楚理解自己需要达到的标准 | 0.85 | 5.15 | 1.29 |

Dimension Reliability: α=0.88, CR=0.89, AVE=0.67

**Dimension 2: Goal Challenge (4 items)**

| Item | English Version | Chinese Version | Loading | M | SD |
| --- | --- | --- | --- | --- | --- |
| GS5 | The goals set for me are appropriately challenging | 为我设定的目标具有适当的挑战性 | 0.78 | 5.08 | 1.35 |
| GS6 | Achieving my goals requires me to stretch my abilities | 实现目标需要我充分发挥自己的能力 | 0.81 | 5.21 | 1.31 |
| GS7 | My goals push me to improve beyond my current level | 我的目标促使我在现有水平上取得进步 | 0.79 | 5.18 | 1.28 |
| GS8 | The difficulty level of my goals is neither too easy nor too hard | 我的目标难度既不太容易也不太困难 | 0.74 | 5.02 | 1.38 |

Dimension Reliability: α=0.85, CR=0.86, AVE=0.61

**Dimension 3: Goal Acceptance (4 items)**

| Item | English Version | Chinese Version | Loading | M | SD |
| --- | --- | --- | --- | --- | --- |
| GS9 | I fully accept and identify with the established goals | 我完全接受并认同既定的目标 | 0.83 | 5.26 | 1.19 |
| GS10 | I believe these goals are meaningful and worthwhile | 我相信这些目标是有意义和有价值的 | 0.86 | 5.33 | 1.16 |
| GS11 | I am committed to achieving the goals that have been set | 我致力于实现已设定的目标 | 0.85 | 5.29 | 1.21 |
| GS12 | The goals align well with my personal values and aspirations | 这些目标与我的个人价值观和愿望高度一致 | 0.80 | 5.11 | 1.26 |

Dimension Reliability: α=0.87, CR=0.88, AVE=0.64

**Dimension 4: Feedback Mechanism (4 items)**

| Item | English Version | Chinese Version | Loading | M | SD |
| --- | --- | --- | --- | --- | --- |
| GS13 | I regularly receive feedback on my progress toward goals | 我定期收到关于目标进展的反馈 | 0.82 | 5.19 | 1.25 |
| GS14 | The feedback I receive helps me understand how well I am doing | 我收到的反馈帮助我了解自己做得如何 | 0.84 | 5.24 | 1.22 |
| GS15 | I get timely information about whether I am on track | 我能及时获得关于是否按计划进行的信息 | 0.81 | 5.16 | 1.28 |
| GS16 | Feedback is provided in a way that helps me improve | 反馈的提供方式有助于我改进 | 0.79 | 5.21 | 1.24 |

Dimension Reliability: α=0.86, CR=0.87, AVE=0.62

Total Scale Reliability: α=0.91, CR=0.92, AVE=0.65 CFA Model Fit: χ²/df=2.18, CFI=0.94, TLI=0.93, RMSEA=0.058 (90%CI[0.051,0.065]), SRMR=0.047

**Part B: Student Learning Behavior Scale (12 items)**

**Dimension 1: Learning Engagement (4 items)**

| Item | English Version | Chinese Version | Loading | M | SD |
| --- | --- | --- | --- | --- | --- |
| SB1 | I actively participate in class discussions and activities | 我积极参与课堂讨论和活动 | 0.83 | 4.95 | 1.32 |
| SB2 | I pay close attention during lessons | 我在课堂上全神贯注 | 0.81 | 5.02 | 1.28 |
| SB3 | I am enthusiastic about learning new things | 我对学习新知识充满热情 | 0.85 | 4.88 | 1.35 |
| SB4 | I ask questions when I don't understand something | 我在不理解时会提出问题 | 0.78 | 4.79 | 1.41 |

Dimension Reliability: α=0.87, CR=0.88, AVE=0.65

**Dimension 2: Learning Persistence (4 items)**

| Item | English Version | Chinese Version | Loading | M | SD |
| --- | --- | --- | --- | --- | --- |
| SB5 | I persist in completing difficult assignments even when frustrated | 即使遇到挫折，我也会坚持完成困难的作业 | 0.82 | 4.84 | 1.38 |
| SB6 | I don't give up easily when learning becomes challenging | 当学习变得具有挑战性时，我不轻易放弃 | 0.86 | 4.91 | 1.33 |
| SB7 | I keep trying different approaches until I succeed | 我会不断尝试不同方法直到成功 | 0.84 | 4.87 | 1.36 |
| SB8 | I continue working on tasks even when they take longer than expected | 即使任务花费的时间超出预期，我也会继续完成 | 0.79 | 4.93 | 1.31 |

Dimension Reliability: α=0.88, CR=0.89, AVE=0.67

**Dimension 3: Learning Strategy Use (4 items)**

| Item | English Version | Chinese Version | Loading | M | SD |
| --- | --- | --- | --- | --- | --- |
| SB9 | I use effective strategies to organize and understand learning materials | 我使用有效的策略来组织和理解学习材料 | 0.81 | 4.76 | 1.35 |
| SB10 | I plan my study time and monitor my progress | 我规划学习时间并监控进展 | 0.83 | 4.82 | 1.32 |
| SB11 | I adjust my learning methods based on what works best | 我根据最有效的方法调整学习策略 | 0.85 | 4.89 | 1.28 |
| SB12 | I connect new information with what I already know | 我将新信息与已知知识联系起来 | 0.78 | 4.91 | 1.30 |

Dimension Reliability: α=0.86, CR=0.87, AVE=0.63

Total Scale Reliability: α=0.89, CR=0.90, AVE=0.64 CFA Model Fit: χ²/df=2.05, CFI=0.95, TLI=0.94, RMSEA=0.054 (90%CI[0.047,0.061]), SRMR=0.045

**Part C: Learning Motivation Scale (10 items)**

**Dimension 1: Intrinsic Motivation (5 items)**

| Item | English Version | Chinese Version | Loading | M | SD |
| --- | --- | --- | --- | --- | --- |
| LM1 | I study because I enjoy learning new things | 我学习是因为我喜欢学习新知识 | 0.84 | 4.68 | 1.42 |
| LM2 | Learning itself is rewarding for me | 学习本身对我来说是有意义的 | 0.86 | 4.72 | 1.38 |
| LM3 | I am curious about the subjects I study | 我对所学科目感到好奇 | 0.82 | 4.79 | 1.36 |
| LM4 | I study because it is personally meaningful to me | 我学习是因为这对我个人有意义 | 0.85 | 4.73 | 1.39 |
| LM5 | I find satisfaction in understanding complex concepts | 我在理解复杂概念时感到满足 | 0.81 | 4.65 | 1.44 |

Dimension Reliability: α=0.89, CR=0.90, AVE=0.68

**Dimension 2: Extrinsic Motivation (5 items)**

| Item | English Version | Chinese Version | Loading | M | SD |
| --- | --- | --- | --- | --- | --- |
| LM6 | I study to get good grades | 我学习是为了取得好成绩 | 0.78 | 5.12 | 1.25 |
| LM7 | I want to please my teachers/parents | 我想让老师/父母满意 | 0.76 | 4.85 | 1.33 |
| LM8 | I study because it will help me get a good job | 我学习是因为这将帮助我找到好工作 | 0.82 | 4.93 | 1.29 |
| LM9 | I need to prove to myself and others that I can succeed | 我需要向自己和他人证明我能成功 | 0.79 | 4.71 | 1.38 |
| LM10 | External rewards motivate me to study harder | 外部奖励激励我更努力学习 | 0.74 | 4.68 | 1.41 |

Dimension Reliability: α=0.84, CR=0.85, AVE=0.59

Total Scale Reliability: α=0.88, CR=0.89, AVE=0.62 CFA Model Fit: χ²/df=2.12, CFI=0.94, TLI=0.93, RMSEA=0.056 (90%CI[0.049,0.063]), SRMR=0.048

**Part D: Self-Efficacy Scale (8 items)**

| Item | English Version | Chinese Version | Loading | M | SD |
| --- | --- | --- | --- | --- | --- |
| SE1 | I am confident I can master the most difficult materials in my courses | 我有信心掌握课程中最困难的材料 | 0.82 | 4.87 | 1.28 |
| SE2 | I believe I can succeed in achieving my learning goals | 我相信我能成功实现学习目标 | 0.85 | 4.96 | 1.22 |
| SE3 | I am capable of learning even the most challenging content | 即使是最具挑战性的内容，我也有能力学习 | 0.83 | 4.89 | 1.26 |
| SE4 | I can perform well on tests and assignments | 我能在考试和作业中表现良好 | 0.81 | 5.01 | 1.19 |
| SE5 | I have the skills needed to succeed academically | 我拥有在学业上取得成功所需的技能 | 0.84 | 4.93 | 1.23 |
| SE6 | I can overcome obstacles that affect my learning | 我能克服影响学习的障碍 | 0.79 | 4.85 | 1.28 |
| SE7 | I am confident in my ability to understand complex ideas | 我对自己理解复杂概念的能力有信心 | 0.82 | 4.91 | 1.24 |
| SE8 | I trust my ability to achieve academic excellence | 我相信自己有能力取得学业优异成绩 | 0.80 | 4.88 | 1.27 |

Scale Reliability: α=0.86, CR=0.87, AVE=0.61 CFA Model Fit: χ²/df=1.98, CFI=0.96, TLI=0.95, RMSEA=0.052 (90%CI[0.044,0.060]), SRMR=0.043

**Part E: Teacher Teaching Behavior Scale (14 items)**

**Dimension 1: Teaching Innovation (5 items)**

| Item | English Version | Chinese Version | Loading | M | SD |
| --- | --- | --- | --- | --- | --- |
| TB1 | I actively try new teaching methods and approaches | 我积极尝试新的教学方法和途径 | 0.82 | 5.08 | 1.22 |
| TB2 | I incorporate innovative techniques into my lessons | 我将创新技术融入课堂教学 | 0.84 | 5.15 | 1.18 |
| TB3 | I am willing to experiment with different instructional strategies | 我愿意尝试不同的教学策略 | 0.81 | 5.12 | 1.20 |
| TB4 | I seek out and apply new ideas in my teaching | 我寻找并应用新的教学理念 | 0.79 | 5.03 | 1.25 |
| TB5 | I continuously look for ways to improve my teaching practices | 我不断寻找改进教学实践的方法 | 0.83 | 5.21 | 1.16 |

Dimension Reliability: α=0.88, CR=0.89, AVE=0.65

**Dimension 2: Teaching Engagement (5 items)**

| Item | English Version | Chinese Version | Loading | M | SD |
| --- | --- | --- | --- | --- | --- |
| TB6 | I invest substantial time and energy in lesson preparation | 我在备课上投入大量时间和精力 | 0.85 | 5.19 | 1.17 |
| TB7 | I am fully committed to my students' learning | 我全心投入学生的学习 | 0.87 | 5.24 | 1.14 |
| TB8 | I dedicate myself to creating quality learning experiences | 我致力于创造高质量的学习体验 | 0.84 | 5.16 | 1.19 |
| TB9 | I actively participate in professional development activities | 我积极参与专业发展活动 | 0.79 | 5.08 | 1.23 |
| TB10 | I go above and beyond basic teaching requirements | 我的工作超出了基本教学要求 | 0.81 | 5.11 | 1.20 |

Dimension Reliability: α=0.89, CR=0.90, AVE=0.67

**Dimension 3: Effectiveness Orientation (4 items)**

| Item | English Version | Chinese Version | Loading | M | SD |
| --- | --- | --- | --- | --- | --- |
| TB11 | I continuously adjust teaching strategies to improve student learning outcomes | 我不断调整教学策略以提高学生学习成果 | 0.83 | 5.13 | 1.18 |
| TB12 | I assess and respond to students' learning needs | 我评估并回应学生的学习需求 | 0.85 | 5.17 | 1.16 |
| TB13 | I use data and feedback to enhance teaching effectiveness | 我使用数据和反馈来提高教学效果 | 0.82 | 5.06 | 1.21 |
| TB14 | I focus on achieving measurable learning outcomes | 我专注于实现可衡量的学习成果 | 0.80 | 5.09 | 1.19 |

Dimension Reliability: α=0.87, CR=0.88, AVE=0.63

Total Scale Reliability: α=0.87, CR=0.88, AVE=0.62 CFA Model Fit: χ²/df=2.12, CFI=0.96, TLI=0.95, RMSEA=0.056 (90%CI[0.049,0.063]), SRMR=0.044

**Part F: Professional Identity Scale (12 items)**

**Dimension 1: Value Identification (4 items)**

| Item | English Version | Chinese Version | Loading | M | SD |
| --- | --- | --- | --- | --- | --- |
| PI1 | I believe teaching is a meaningful and valuable profession | 我认为教学是一个有意义和有价值的职业 | 0.86 | 5.35 | 1.08 |
| PI2 | The values of the teaching profession align with my personal values | 教学职业的价值观与我的个人价值观一致 | 0.84 | 5.31 | 1.11 |
| PI3 | I am proud to be a teacher | 我为成为一名教师感到自豪 | 0.87 | 5.38 | 1.05 |
| PI4 | Teaching fulfills my sense of purpose | 教学实现了我的人生目标 | 0.82 | 5.24 | 1.13 |

Dimension Reliability: α=0.89, CR=0.90, AVE=0.69

**Dimension 2: Emotional Attachment (4 items)**

| Item | English Version | Chinese Version | Loading | M | SD |
| --- | --- | --- | --- | --- | --- |
| PI5 | I have a strong emotional connection to the teaching profession | 我对教学职业有强烈的情感联系 | 0.83 | 5.26 | 1.09 |
| PI6 | I feel a sense of belonging in the education community | 我在教育群体中有归属感 | 0.85 | 5.29 | 1.07 |
| PI7 | My identity as a teacher is important to who I am | 作为教师的身份对我的自我认同很重要 | 0.84 | 5.27 | 1.08 |
| PI8 | I care deeply about the teaching profession | 我深切关心教学事业 | 0.82 | 5.32 | 1.06 |

Dimension Reliability: α=0.88, CR=0.89, AVE=0.67

**Dimension 3: Behavioral Tendency (4 items)**

| Item | English Version | Chinese Version | Loading | M | SD |
| --- | --- | --- | --- | --- | --- |
| PI9 | I actively promote the teaching profession to others | 我向他人积极宣传教学职业 | 0.79 | 5.18 | 1.15 |
| PI10 | I plan to remain in the teaching profession long-term | 我计划长期从事教学职业 | 0.81 | 5.24 | 1.12 |
| PI11 | I advocate for the interests of teachers and students | 我为教师和学生的利益发声 | 0.80 | 5.21 | 1.14 |
| PI12 | I contribute to the development of the teaching profession | 我为教学职业的发展做出贡献 | 0.78 | 5.19 | 1.16 |

Dimension Reliability: α=0.85, CR=0.86, AVE=0.61

Total Scale Reliability: α=0.90, CR=0.91, AVE=0.66 CFA Model Fit: χ²/df=2.08, CFI=0.96, TLI=0.95, RMSEA=0.055 (90%CI[0.047,0.062]), SRMR=0.046

**Part G: Teaching Efficacy Scale (15 items)**

**Dimension 1: Instructional Strategy Efficacy (5 items)**

| Item | English Version | Chinese Version | Loading | M | SD |
| --- | --- | --- | --- | --- | --- |
| TE1 | I can use a variety of instructional strategies effectively | 我能有效使用各种教学策略 | 0.84 | 5.18 | 1.12 |
| TE2 | I know how to design lessons that meet diverse learning needs | 我知道如何设计满足不同学习需求的课程 | 0.86 | 5.21 | 1.09 |
| TE3 | I am skilled at explaining difficult concepts in understandable ways | 我擅长用易懂的方式解释难懂的概念 | 0.83 | 5.15 | 1.14 |
| TE4 | I can adapt my teaching to different types of learners | 我能根据不同类型的学习者调整教学 | 0.82 | 5.13 | 1.15 |
| TE5 | I am confident in my ability to implement effective teaching methods | 我对自己实施有效教学方法的能力有信心 | 0.85 | 5.19 | 1.11 |

Dimension Reliability: α=0.90, CR=0.91, AVE=0.69

**Dimension 2: Classroom Management Efficacy (5 items)**

| Item | English Version | Chinese Version | Loading | M | SD |
| --- | --- | --- | --- | --- | --- |
| TE6 | I can maintain order and discipline in my classroom | 我能维持课堂秩序和纪律 | 0.82 | 5.16 | 1.13 |
| TE7 | I am effective at managing classroom behavior | 我能有效管理课堂行为 | 0.84 | 5.19 | 1.11 |
| TE8 | I can create a positive learning environment | 我能创造积极的学习环境 | 0.85 | 5.23 | 1.08 |
| TE9 | I handle disruptions effectively without losing instructional time | 我能有效处理干扰而不损失教学时间 | 0.80 | 5.11 | 1.16 |
| TE10 | I am confident in establishing classroom routines and procedures | 我对建立课堂常规和程序有信心 | 0.83 | 5.17 | 1.12 |

Dimension Reliability: α=0.89, CR=0.90, AVE=0.67

**Dimension 3: Student Engagement Efficacy (5 items)**

| Item | English Version | Chinese Version | Loading | M | SD |
| --- | --- | --- | --- | --- | --- |
| TE11 | I can motivate students who show low interest in learning | 我能激励对学习兴趣不高的学生 | 0.81 | 5.08 | 1.17 |
| TE12 | I am effective at getting students to participate actively | 我能有效让学生积极参与 | 0.83 | 5.14 | 1.13 |
| TE13 | I can help students believe they can succeed | 我能帮助学生相信他们能够成功 | 0.84 | 5.18 | 1.10 |
| TE14 | I know how to foster students' curiosity and love of learning | 我知道如何培养学生的好奇心和学习热爱 | 0.82 | 5.12 | 1.14 |
| TE15 | I am confident in my ability to engage all students in learning | 我对自己让所有学生参与学习的能力有信心 | 0.80 | 5.09 | 1.16 |

Dimension Reliability: α=0.88, CR=0.89, AVE=0.65

Total Scale Reliability: α=0.92, CR=0.93, AVE=0.68 CFA Model Fit: χ²/df=2.15, CFI=0.96, TLI=0.95, RMSEA=0.057 (90%CI[0.050,0.064]), SRMR=0.045

**Part H: Organizational Support Scale (18 items)**

**Dimension 1: Institutional Support (6 items)**

| Item | English Version | Chinese Version | Loading | M | SD |
| --- | --- | --- | --- | --- | --- |
| OS1 | The school has clear policies supporting goal achievement | 学校有明确的政策支持目标实现 | 0.82 | 4.72 | 1.35 |
| OS2 | Performance evaluation systems are fair and transparent | 绩效评估系统公平透明 | 0.84 | 4.68 | 1.38 |
| OS3 | The school provides recognition for goal accomplishment | 学校对目标完成提供认可 | 0.81 | 4.65 | 1.41 |
| OS4 | Institutional procedures facilitate rather than hinder goal pursuit | 制度程序促进而非阻碍目标追求 | 0.79 | 4.59 | 1.43 |
| OS5 | The school's reward system aligns with goal achievement | 学校的奖励制度与目标实现相一致 | 0.83 | 4.63 | 1.39 |
| OS6 | Administrative support is readily available when needed | 需要时能轻易获得行政支持 | 0.80 | 4.71 | 1.36 |

Dimension Reliability: α=0.89, CR=0.90, AVE=0.64

**Dimension 2: Resource Support (6 items)**

| Item | English Version | Chinese Version | Loading | M | SD |
| --- | --- | --- | --- | --- | --- |
| OS7 | The school provides adequate resources for professional development | 学校为专业发展提供充足资源 | 0.83 | 4.76 | 1.34 |
| OS8 | Learning/teaching materials and facilities are sufficient | 学习/教学材料和设施充足 | 0.85 | 4.81 | 1.31 |
| OS9 | Technology and tools needed for success are available | 成功所需的技术和工具是可用的 | 0.82 | 4.73 | 1.36 |
| OS10 | Financial support is provided for goal-related activities | 为目标相关活动提供财务支持 | 0.79 | 4.58 | 1.42 |
| OS11 | Time allocations are reasonable for achieving goals | 实现目标的时间分配是合理的 | 0.81 | 4.67 | 1.38 |
| OS12 | Access to expertise and consultation is readily available | 能轻易获得专业知识和咨询 | 0.80 | 4.69 | 1.37 |

Dimension Reliability: α=0.90, CR=0.91, AVE=0.66

**Dimension 3: Cultural Support (6 items)**

| Item | English Version | Chinese Version | Loading | M | SD |
| --- | --- | --- | --- | --- | --- |
| OS13 | The school culture encourages continuous improvement | 学校文化鼓励持续改进 | 0.84 | 4.79 | 1.32 |
| OS14 | Collaboration and teamwork are valued and promoted | 协作和团队合作受到重视和促进 | 0.86 | 4.83 | 1.29 |
| OS15 | Innovation and creativity are welcomed | 创新和创造力受到欢迎 | 0.83 | 4.75 | 1.34 |
| OS16 | There is a supportive atmosphere for taking on challenges | 接受挑战的氛围支持性强 | 0.82 | 4.71 | 1.36 |
| OS17 | Leaders model goal-oriented behavior | 领导者示范目标导向的行为 | 0.81 | 4.68 | 1.38 |
| OS18 | The organizational climate promotes personal and professional growth | 组织氛围促进个人和专业成长 | 0.85 | 4.77 | 1.33 |

Dimension Reliability: α=0.91, CR=0.92, AVE=0.68

**Supplementary Table S2. Cross-Loadings and Discriminant Validity Matrix**

**Student Sample (N=1,247)**

|  | GS | LM | SE | SB | OS | √AVE |
| --- | --- | --- | --- | --- | --- | --- |
| Goal-Setting (GS) | 0.81 | 0.48 | 0.51 | 0.58 | 0.42 | 0.81 |
| Learning Motivation (LM) | 0.48 | 0.79 | 0.64 | 0.71 | 0.38 | 0.79 |
| Self-Efficacy (SE) | 0.51 | 0.64 | 0.78 | 0.66 | 0.35 | 0.78 |
| Student Behavior (SB) | 0.58 | 0.71 | 0.66 | 0.80 | 0.41 | 0.80 |
| Organizational Support (OS) | 0.42 | 0.38 | 0.35 | 0.41 | 0.82 | 0.82 |

Note. Diagonal elements (bold) are square roots of AVE. Off-diagonal elements are construct correlations. For discriminant validity, diagonal elements should exceed off-diagonal elements in the same row/column.

**Teacher Sample (N=358)**

|  | GS | PI | TE | TB | OS | √AVE |
| --- | --- | --- | --- | --- | --- | --- |
| Goal-Setting (GS) | 0.79 | 0.44 | 0.46 | 0.52 | 0.43 | 0.79 |
| Professional Identity (PI) | 0.44 | 0.81 | 0.61 | 0.67 | 0.45 | 0.81 |
| Teaching Efficacy (TE) | 0.46 | 0.61 | 0.82 | 0.69 | 0.48 | 0.82 |
| Teacher Behavior (TB) | 0.52 | 0.67 | 0.69 | 0.79 | 0.48 | 0.79 |
| Organizational Support (OS) | 0.43 | 0.45 | 0.48 | 0.48 | 0.82 | 0.82 |

Note. All diagonal elements exceed off-diagonal elements, supporting discriminant validity via Fornell-Larcker criterion.

**Supplementary Table S3. Measurement Invariance Testing Results**

**Student Sample Across Educational Stages**

| Model | χ² | df | CFI | TLI | RMSEA | SRMR | Comparison | Δχ² | Δdf | ΔCFI | ΔRMSEA |
| --- | --- | --- | --- | --- | --- | --- | --- | --- | --- | --- | --- |
| Configural | 4,235.67 | 1,848 | 0.941 | 0.936 | 0.054 | 0.061 | — | — | — | — | — |
| Metric | 4,289.34 | 1,896 | 0.940 | 0.937 | 0.053 | 0.063 | Config vs Metric | 53.67 | 48 | -0.001 | -0.001 |
| Scalar | 4,412.18 | 1,944 | 0.937 | 0.936 | 0.054 | 0.065 | Metric vs Scalar | 122.84* | 48 | -0.003 | +0.001 |
| Partial Scalar† | 4,358.91 | 1,932 | 0.939 | 0.937 | 0.053 | 0.064 | Metric vs Partial | 69.57 | 36 | -0.001 | 0.000 |

Note. *p < 0.05. †Partial scalar model freed 12 item intercepts. ΔCFI < 0.010 and ΔRMSEA < 0.015 indicate invariance (Chen, 2007).

**Teacher Sample Across Experience Groups**

| Model | χ² | df | CFI | TLI | RMSEA | SRMR | Comparison | Δχ² | Δdf | ΔCFI | ΔRMSEA |
| --- | --- | --- | --- | --- | --- | --- | --- | --- | --- | --- | --- |
| Configural | 2,847.23 | 1,527 | 0.952 | 0.948 | 0.062 | 0.068 | — | — | — | — | — |
| Metric | 2,891.45 | 1,581 | 0.951 | 0.949 | 0.061 | 0.069 | Config vs Metric | 44.22 | 54 | -0.001 | -0.001 |
| Scalar | 2,985.67 | 1,635 | 0.948 | 0.948 | 0.061 | 0.071 | Metric vs Scalar | 94.22 | 54 | -0.003 | 0.000 |
| Partial Scalar† | 2,941.88 | 1,617 | 0.950 | 0.949 | 0.061 | 0.070 | Metric vs Partial | 50.43 | 36 | -0.001 | 0.000 |

Note. Partial scalar model freed 8 item intercepts.

**Supplementary Table S4. Item-Level Descriptive Statistics by Educational Stage**

**Goal-Setting Mechanism: Goal Clarity Dimension**

| Item | Junior High (n=418) | Senior High (n=426) | University (n=403) | F | p | η² |
| --- | --- | --- | --- | --- | --- | --- |
|  | M (SD) | M (SD) | M (SD) |  |  |  |
| GS1 | 5.08 (1.28) | 5.42 (1.15) | 5.43 (1.08) | 12.34 | <.001 | 0.019 |
| GS2 | 5.02 (1.31) | 5.38 (1.21) | 5.44 (1.12) | 15.67 | <.001 | 0.024 |
| GS3 | 4.95 (1.35) | 5.29 (1.26) | 5.33 (1.18) | 11.89 | <.001 | 0.019 |
| GS4 | 4.89 (1.38) | 5.24 (1.28) | 5.32 (1.20) | 13.45 | <.001 | 0.021 |

Note. One-way ANOVA with post-hoc Tukey tests. Senior high and university students scored significantly higher than junior high students on all items (p < .05). No significant differences between senior high and university students.

**Learning Motivation: Intrinsic vs. Extrinsic by Stage**

| Dimension | Junior High | Senior High | University | F | p | η² |
| --- | --- | --- | --- | --- | --- | --- |
| Intrinsic Motivation | 4.82 (1.38) | 4.58 (1.42) | 4.79 (1.45) | 4.56 | .011 | 0.007 |
| Extrinsic Motivation | 4.98 (1.29) | 5.21 (1.18) | 4.63 (1.37) | 26.78 | <.001 | 0.041 |

Note. Senior high students showed significantly higher extrinsic motivation than both junior high and university students, reflecting high-stakes examination pressure.

Supplementary Table S5. Correlation Matrices by Educational Stage

**Junior High Students (n=418)**

|  | 1 | 2 | 3 | 4 | 5 |
| --- | --- | --- | --- | --- | --- |
| 1. Goal-Setting | — |  |  |  |  |
| 2. Learning Motivation | .44*** | — |  |  |  |
| 3. Self-Efficacy | .47*** | .61*** | — |  |  |
| 4. Student Behavior | .53*** | .68*** | .62*** | — |  |
| 5. Org. Support | .39*** | .35*** | .32*** | .38*** | — |

**Senior High Students (n=426)**

|  | 1 | 2 | 3 | 4 | 5 |
| --- | --- | --- | --- | --- | --- |
| 1. Goal-Setting | — |  |  |  |  |
| 2. Learning Motivation | .51*** | — |  |  |  |
| 3. Self-Efficacy | .54*** | .66*** | — |  |  |
| 4. Student Behavior | .62*** | .74*** | .69*** | — |  |
| 5. Org. Support | .45*** | .41*** | .38*** | .44*** | — |

**University Students (n=403)**

|  | 1 | 2 | 3 | 4 | 5 |
| --- | --- | --- | --- | --- | --- |
| 1. Goal-Setting | — |  |  |  |  |
| 2. Learning Motivation | .49*** | — |  |  |  |
| 3. Self-Efficacy | .52*** | .65*** | — |  |  |
| 4. Student Behavior | .59*** | .72*** | .67*** | — |  |
| 5. Org. Support | .42*** | .37*** | .34*** | .40*** | — |

Note. ***p < .001. Senior high students showed consistently stronger correlations, reflecting higher goal-orientation during this critical academic period.

**Supplementary Table S6. Correlation Matrices by Teacher Experience**

**Novice Teachers (1-5 years, n=89)**

|  | 1 | 2 | 3 | 4 | 5 |
| --- | --- | --- | --- | --- | --- |
| 1. Goal-Setting | — |  |  |  |  |
| 2. Professional Identity | .38** | — |  |  |  |
| 3. Teaching Efficacy | .41*** | .55*** | — |  |  |
| 4. Teacher Behavior | .46*** | .61*** | .64*** | — |  |
| 5. Org. Support | .37** | .39** | .42*** | .43*** | — |

**Mid-Career Teachers (6-15 years, n=210)**

|  | 1 | 2 | 3 | 4 | 5 |
| --- | --- | --- | --- | --- | --- |
| 1. Goal-Setting | — |  |  |  |  |
| 2. Professional Identity | .48*** | — |  |  |  |
| 3. Teaching Efficacy | .51*** | .64*** | — |  |  |
| 4. Teacher Behavior | .57*** | .71*** | .73*** | — |  |
| 5. Org. Support | .47*** | .48*** | .52*** | .52*** | — |

**Senior Teachers (16+ years, n=59)**

|  | 1 | 2 | 3 | 4 | 5 |
| --- | --- | --- | --- | --- | --- |
| 1. Goal-Setting | — |  |  |  |  |
| 2. Professional Identity | .42** | — |  |  |  |
| 3. Teaching Efficacy | .44*** | .59*** | — |  |  |
| 4. Teacher Behavior | .49*** | .65*** | .67*** | — |  |
| 5. Org. Support | .40** | .43** | .46*** | .45*** | — |

Note. **p < .01, ***p < .001. Mid-career teachers showed strongest correlations across all variables, supporting the finding that this group is most responsive to goal-setting mechanisms.

**Supplementary Table S7. Regional Comparison of Key Variables**

**Student Sample**

| Variable | Eastern (n=415) | Central (n=416) | Western (n=416) | F | p | η² |
| --- | --- | --- | --- | --- | --- | --- |
|  | M (SD) | M (SD) | M (SD) |  |  |  |
| Goal-Setting | 5.31 (1.10) | 5.22 (1.15) | 5.16 (1.18) | 2.18 | .113 | 0.004 |
| Learning Motivation | 4.82 (1.29) | 4.73 (1.35) | 4.72 (1.36) | 0.89 | .411 | 0.001 |
| Self-Efficacy | 5.01 (1.15) | 4.91 (1.21) | 4.87 (1.22) | 1.87 | .154 | 0.003 |
| Student Behavior | 4.96 (1.22) | 4.87 (1.28) | 4.84 (1.29) | 1.23 | .293 | 0.002 |
| Org. Support | 4.79 (1.24) | 4.65 (1.30) | 4.61 (1.31) | 2.67 | .069 | 0.004 |

**Teacher Sample**

| Variable | Eastern (n=119) | Central (n=125) | Western (n=114) | F | p | η² |
| --- | --- | --- | --- | --- | --- | --- |
| Goal-Setting | 5.48 (1.05) | 5.39 (1.08) | 5.35 (1.12) | 0.64 | .528 | 0.004 |
| Professional Identity | 5.34 (0.98) | 5.27 (1.03) | 5.22 (1.06) | 0.58 | .559 | 0.003 |
| Teaching Efficacy | 5.21 (1.04) | 5.13 (1.08) | 5.11 (1.09) | 0.42 | .658 | 0.002 |
| Teacher Behavior | 5.18 (1.12) | 5.11 (1.16) | 5.07 (1.17) | 0.39 | .677 | 0.002 |
| Org. Support | 4.93 (1.18) | 4.82 (1.22) | 4.79 (1.24) | 0.61 | .543 | 0.003 |

Note. No significant regional differences were found, supporting the cross-regional stability of the theoretical model. This justifies pooling samples across regions for main analyses.

**Supplementary Table S8. Internal Consistency Reliability by Subgroups**

**Student Sample: Cronbach's Alpha by Educational Stage**

| Scale | Junior High | Senior High | University | Overall |
| --- | --- | --- | --- | --- |
| Goal-Setting Mechanism | .89 | .92 | .91 | .91 |
| Learning Motivation | .86 | .90 | .88 | .88 |
| Self-Efficacy | .84 | .87 | .86 | .86 |
| Student Behavior | .87 | .91 | .89 | .89 |
| Organizational Support | .92 | .94 | .93 | .93 |

**Teacher Sample: Cronbach's Alpha by Experience Level**

| Scale | 1-5 Years | 6-15 Years | 16+ Years | Overall |
| --- | --- | --- | --- | --- |
| Goal-Setting Mechanism | .87 | .90 | .88 | .89 |
| Professional Identity | .88 | .91 | .90 | .90 |
| Teaching Efficacy | .90 | .93 | .91 | .92 |
| Teacher Behavior | .85 | .88 | .86 | .87 |
| Organizational Support | .93 | .95 | .94 | .94 |

Note. All reliability coefficients exceed 0.80 threshold across all subgroups, indicating stable and consistent measurement across diverse populations.
